# Prehospital qSOFA Is Associated with 30-Day Mortality Within Emergency Department Triage Strata in Ambulance-Transported Adults with Suspected Infection: An Exploratory Three-Year Retrospective Cohort Study

**DOI:** 10.3390/jcm15197591

**Published:** 2026-09-30

**Authors:** Ayşenur Özçelik, Faruk Danış, Kaan Çelik

**Affiliations:** 1 Department of Emergency Medicine, Faculty of Medicine, Bolu Abant İzzet Baysal University, 14030 Bolu, Türkiye; aysenur_ozcelik@hotmail.com (A.Ö.); kaan.celik@ibu.edu.tr (K.Ç.); 2 Department of Emergency Medicine, Bolu İzzet Baysal Training and Research Hospital, 14030 Bolu, Türkiye

**Keywords:** sepsis, quick sequential organ failure assessment, emergency medical services, prehospital emergency care, triage, risk assessment, hospital mortality, organ dysfunction scores

## Abstract

**Background/Objectives**: Prehospital quick Sequential Organ Failure Assessment (qSOFA) screens poorly for sepsis, but an already-recorded field score may still inform the receiving physician. We asked whether it is associated with 30-day mortality within each emergency department (ED) triage classification, and what it adds to the arrival score. Methods: This study focused on a retrospective cohort of 1077 adults with suspected infection transported by ambulance to one Turkish university hospital ED, 2022 to 2024 (42.1% of 2558 eligible; most exclusions arose because the hospital episode record could not be retrieved). Patients were stratified by ED-arrival qSOFA classification (2 or more), then by prehospital classification. The primary outcome was 30-day mortality from hospital and national death records. The stratified analysis was post hoc. Results: Among 685 triage-negative patients, 30-day mortality was 13.9% when the field score had been below 2 and 26.0% when it had been 2 or more (adjusted odds ratio 2.06, 95% CI 1.28 to 3.25); among 392 triage-positive patients, 40.4% and 55.2% (1.84, 1.23 to 2.76), with no evidence of heterogeneity between strata (interaction odds ratio 0.85, 0.46 to 1.58). Adding the field value to the arrival value raised the area under the curve from 0.682 to 0.713. Added to a model of age, sex, comorbidity, lactate, oxygen saturation, heart rate, and temperature, it remained associated with death (1.71, 1.24 to 2.34) but changed the area under the curve by 0.007 (*p* = 0.23). In 27 selection scenarios returning the 1481 excluded patients, only a reversed association among them could nullify the triage-negative association, whereas the triage-positive association was nullified in 17 scenarios, 4 of them by a positive association. **Conclusions**: In this highly selected single-centre cohort, the prehospital qSOFA carried prognostic information not captured by the arrival score, with small incremental discrimination beyond routine ED data. The findings support preserving prehospital physiology at handover and justify prospective validation, not a change in disposition decisions.

## 1. Introduction

Sepsis remains one of the largest contributors to global mortality. The Global Burden of Disease analysis estimated 48.9 million incident cases and 11.0 million sepsis-related deaths worldwide in 2017, which is 19.7% of all deaths recorded that year [1]. The Sepsis-3 consensus reframed the syndrome as life-threatening organ dysfunction caused by a dysregulated host response to infection [2]. Alongside that definition, the quick Sequential Organ Failure Assessment (qSOFA) score was derived as a bedside prompt [3]. It has three items. One point each is given for altered mentation, a systolic blood pressure of 100 mmHg or less, and a respiratory rate of 22 breaths/min or more.

The score is easy to compute and needs no laboratory result, so emergency medical services (EMS) systems adopted it quickly. Its performance as a screening test has been disappointing. In the emergency department (ED), qSOFA of 2 or more separates in-hospital mortality clearly [4,5]. Its sensitivity is the problem. Pooled sensitivity for mortality is only 60.8% (95% CI 51.4 to 69.4) [6], two-thirds of patients with severe sepsis are missed [7], and sensitivity at triage for patients who later die is around one third [8]. Patients who screen negative are not a low-risk group: 7% of ED patients with suspected infection and qSOFA below 2 went on to develop sepsis [9]. Performance in the field is worse still. Across 221,429 EMS records, prehospital qSOFA had the lowest sensitivity of the tools evaluated, 23.1% [10]. Reported prehospital sensitivities range from 16.3% [11] to 36.3% [12], and no single field strategy reaches both high sensitivity and high specificity [13,14]. In more than 100,000 ambulance patients, qSOFA discriminated 30-day mortality less well than NEWS2 and other aggregate scores [15].

Recognition itself remains uncommon. Prehospital sepsis recognition occurred in 18% of 20,172 EMS-transported patients later given a sepsis diagnosis [16]. A recent single-centre series put the same problem in concrete terms: EMS crews recorded a working diagnosis of suspected sepsis in a minority of the patients who met criteria on arrival, and the field scores computed retrospectively did not close the gap [17]. Where the field score is recorded, it does separate outcomes: in-hospital mortality was 14.0% in prehospital qSOFA-positive and 6.0% in prehospital qSOFA-negative patients with suspected infection [18].

The 2026 Surviving Sepsis Campaign guideline reflects that evidence with some precision. It suggests using a standard sepsis screening tool in acutely ill adults travelling to hospital by ambulance, as a conditional recommendation on very low certainty evidence. Separately, it recommends NEWS, NEWS2, MEWS, or SIRS over qSOFA as a single screening tool inside the hospital, and that recommendation is strong, on moderate certainty evidence. The panel did not name a preferred prehospital instrument, and it recorded qSOFA as having the lowest prehospital sepsis sensitivity of the candidates it reviewed [19].

Two features of that position are easy to miss. The strong recommendation against qSOFA concerns its use as a single screening tool, and screening is only one thing a recorded number can do. Nothing in the guideline addresses what an already-documented field score is worth to the physician who later receives the patient. That second question is different, and it is the one an emergency physician actually faces. A patient arrives by ambulance, the triage qSOFA is computed, and the crew’s handover sheet carries a number recorded twenty or forty minutes earlier. What that earlier number is worth has not been established.

There is reason to think it is worth something. Repeated measurement of qSOFA inside the hospital predicts mortality better than a single reading. In 37,591 encounters, the 48 h mean reached an AUROC of 0.86 (95% CI 0.85 to 0.86) against 0.79 (0.78 to 0.80) for the initial score [20]. A qSOFA that rises over the first three ED hours carries 44% in-hospital mortality against 18% when it falls [21], although serial measurement every 30 min added little in a smaller cohort [22]. Across the prehospital-to-hospital interval specifically, evidence is thinner and comes mostly from adjacent populations. In 90,974 trauma patients, a prehospital qSOFA of 0 that had become 2 or more on arrival carried an adjusted odds ratio of 3.46 (95% CI 2.41 to 4.85) for in-hospital death [23]. Tracking NEWS2 from scene to ED triage improved two-day mortality discrimination across the same interval [24]. Among 86,069 EMS-transported non-trauma patients classified as low risk at ED triage, in-hospital mortality rose from 1.2% to 2.4% to 6.8% across low, medium, and high prehospital NEWS categories [25]. Closest to our question, a combined field-and-department qSOFA identified 44 of 369 sepsis cases (12%) that the ED score alone would have missed in 2407 ambulance-transported adults, even though the two scores had statistically indistinguishable c-statistics [26].

What has not been established is whether the field qSOFA is associated with outcome within a known ED triage classification, for patient-centred outcomes, in a non-trauma population with suspected infection, and whether it adds anything to the arrival score. The prior work differs from that question in one respect or another. It used a different score [24,25], a trauma population [23], a case identification outcome rather than death or organ support [26], or measurements taken entirely inside the hospital [20,21,22]. We therefore asked two linked questions in adults transported to the ED by EMS with suspected infection. Is the prehospital qSOFA classification recorded by the ambulance crew associated with 30-day mortality and organ support outcomes within each ED-arrival qSOFA stratum? And does adding it to the arrival score improve prediction of death?

## 2. Materials and Methods

### 2.1. Study Design and Setting

This was a retrospective observational cohort study conducted at the ED of Bolu Abant İzzet Baysal University Faculty of Medicine, a tertiary referral hospital serving a province of approximately 320,000 people in northwestern Türkiye. All emergency ambulance transport in the province is provided by the national 112 EMS system, which is publicly funded and staffed by paramedics and emergency medical technicians. The study period ran from 1 January 2022 to 31 December 2024. Reporting follows the STROBE statement for observational studies [27], and the secondary diagnostic accuracy component follows STARD 2015 [28]. Data were abstracted from the hospital information management system and from the provincial 112 EMS records onto a predefined form by a single investigator (A.Ö.), who also determined eligibility. The investigator was not blinded to outcome. No second abstractor reviewed the records, so no inter-rater agreement statistic is available; we return to this in the Limitations Section. The data for this study were obtained from the same patient cohort as a residency (medical specialty) thesis completed at the Department of Emergency Medicine, Faculty of Medicine, Bolu Abant İzzet Baysal University.

### 2.2. Participants

Adults aged 18 years or older who were transported to the study ED by 112 EMS during the study period were screened (*n* = 17,786). Eligibility required suspected infection at the time of ED evaluation. This was operationalized retrospectively from the ED record as at least one of five findings: fever above 38 °C or hypothermia below 36 °C; hypotension, tachycardia, or tachypnea; acute altered mental status; a recognised immunosuppressive state; or a severely impaired general condition. Three of these overlap with the physiological items of qSOFA, which we note as a source of selection on the exposure. Eligibility, and the exclusions described next, were adjudicated by one investigator from the ED record; there was no second assessor.

Patients were excluded at screening when a non-infectious process dominated the presentation. That category was broad. It covered trauma, burns, poisoning, acute cardiac and neurologic emergencies, and gastrointestinal, obstetric, psychiatric, and forensic presentations. Patients who had received prehospital advanced life support, defined in this dataset as endotracheal intubation or return of spontaneous circulation after cardiac arrest before hospital arrival, were also excluded, because such treatment alters the vital signs from which the score is built. The number of patients removed under each screening criterion, including advanced life support, was not recorded separately; 15,228 patients were removed at this stage in total, and 2558 met the eligibility criteria.

**Figure 1:**
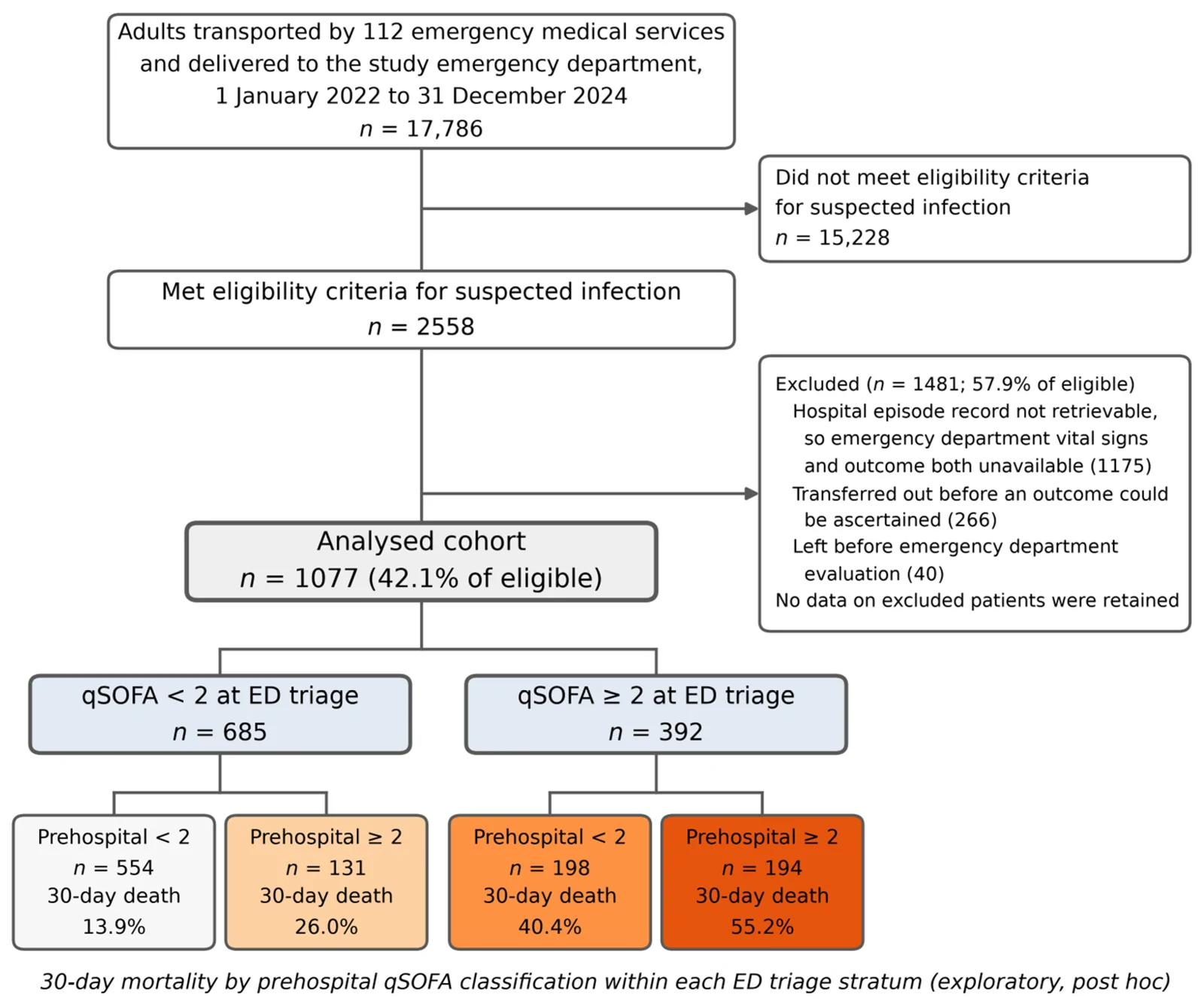
Participant flow and derivation of the four analysis cells. Patients are stratified first by their qSOFA classification at emergency department triage and then by their prehospital classification. The 30-day mortality observed in each of the four cells is shown beneath its label. The three reasons for exclusion after eligibility are shown separately; no data on excluded patients were retained. ED, emergency department; EMS, emergency medical services; qSOFA, quick Sequential Organ Failure Assessment.

A second stage of exclusion followed. It was large, and the reason differed between the three groups that make it up. Of the 2558 eligible patients, 1481 (57.9%) did not enter the analysis. For 1175 of them (45.9% of the eligible cohort), the hospital episode record could not be retrieved from the information system or had not been completed, so neither the vital signs recorded at emergency department triage nor the disposition nor 30-day vital status existed for these patients. Because the arrival vital signs are what define the ED-arrival qSOFA, these patients lacked one of the two measurements the primary analysis compares, as well as its outcome. A further 266 patients (10.4%) were transferred to another institution before an outcome could be ascertained, and 40 (1.6%) left the emergency department before evaluation and so have no arrival measurement either. The exclusion was therefore applied at the level of the record, not at the level of individual variables: laboratory values were absent in these patients, but their absence was a consequence of the missing record rather than the criterion that removed them. No data on excluded patients were retained in the study database, and the ethical approval covers analysis of that de-identified dataset only, so included and excluded patients cannot be compared. We treat this as the principal limitation of the study and quantify its possible effect in Section 3.7. Participant flow, with the three reasons separated, is shown in Figure 1.

### 2.3. Measurements

Prehospital qSOFA was computed from the first set of vital signs documented by the EMS crew at the scene. ED-arrival qSOFA was computed from the first set of vital signs documented at triage. Both scores were re-derived from their components rather than taken from the recorded totals. The conventional thresholds were applied: respiratory rate 22 breaths/min or more, systolic blood pressure 100 mmHg or less, and altered mentation defined as a Glasgow Coma Scale score below 15. The re-derived totals matched the recorded totals in 1077 of 1077 patients for both timepoints. Setting altered mentation at a Glasgow Coma Scale score of 13 or less instead produced 205 disagreements, which establishes the threshold used by the source records.

One constraint of the source data must be stated here. The prehospital respiratory and consciousness fields exist only as the dichotomous criteria, met or not met. No continuous prehospital respiratory rate or Glasgow Coma Scale value is recorded anywhere in the dataset. Of the three field items, only systolic blood pressure survives as a number.

Each patient was assigned to one of four cells. Patients were stratified first by their ED-arrival classification at the conventional threshold of 2 or more, then divided within each stratum by their prehospital classification at the same threshold. This produced a triage-negative stratum containing prehospital-negative and prehospital-positive patients, and a triage-positive stratum containing the same two groups.

Comorbidity burden was the count of nine recorded conditions: hypertension, diabetes mellitus, coronary artery disease, chronic obstructive pulmonary disease, heart failure, chronic kidney disease, malignancy, liver disease, and cerebrovascular disease. A weighted index such as the Charlson score could not be constructed. The dataset does not carry the required severity items. Sepsis-3 status and the SOFA score were adjudicated from ED data alone.

### 2.4. Outcomes

The primary outcome was all-cause mortality within 30 days of ED presentation. Vital status came from two sources. In-hospital deaths and return visits were taken from the hospital information management system. Deaths after discharge were identified through the national civil registration and personal health record systems (MERNİS and e-Nabız), which record every death notified in Türkiye regardless of where it occurred, queried by national identity number. Vital status at day 30 was therefore established for all 1077 analysed patients, including those discharged from the ED or the ward before day 30. The date of death was not extracted, so only 30-day status is available and no time-to-event analysis was possible. Patients whose 30-day status could not be established, chiefly those transferred to another institution, were among the excluded. Secondary outcomes were tracheal intubation, vasopressor use in the ED, vasopressor use on the ward, admission to an intensive care unit (ICU), and a composite organ support outcome defined as any of intubation, vasopressor use, or ICU admission. Total hospital length of stay was also recorded. Vasopressor variables were documented as intensity categories. They were dichotomised as any use versus none, because neither dose nor agent is recorded. ICU admission was derived from the recorded disposition; patients who died in the ED were not counted as ICU admissions. Sepsis-3 status served as the reference standard for a secondary diagnostic accuracy analysis, which is reported as a demoted secondary because the qSOFA items overlap the SOFA domains that define it.

### 2.5. Statistical Analysis

Analyses were performed in IBM SPSS Statistics for Macintosh, version 29.0 (IBM Corp., Armonk, NY, USA), and in R version 4.6.1 (R Foundation for Statistical Computing, Vienna, Austria) within RStudio version 2026.09.0+174 (Posit Software, PBC, Boston, MA, USA). Descriptive statistics and group comparisons were run in SPSS; modelling, bootstrap internal validation, and reclassification analyses were run in R (packages pROC 1.19.0.1, rms 8.1.1, mice 3.19.0, psych 2.6.5, DescTools 0.99.60, nricens 1.6, and dcurves 0.5.1). The analysis code is available from the corresponding author on request. It produced every quantity reported here. All resampling used a fixed seed of 20260902. Every bootstrap ran 1000 resamples. Analyses were defined on nested analysis sets, and each set is reported with its own denominator (Supplementary Table S11). The primary analysis requires the prehospital classification, the ED-arrival classification, and 30-day vital status, which were present in all 1077 analysed patients; adjusted models add age, sex, and comorbidity burden and fit on 1076 because one comorbidity count is missing. Analyses that need the SOFA score or the Sepsis-3 reference standard are secondary and are reported on the subset carrying those variables. No patient contributing to the primary analysis was removed because a laboratory value was missing.

Shapiro–Wilk testing within each of the four cells returned *p* < 0.05 for every continuous variable examined. All continuous variables are therefore summarised as medians with interquartile ranges and compared by Kruskal–Wallis across the four cells and by Mann–Whitney within each stratum. Categorical variables were compared by Pearson chi-square, or by Fisher’s exact test when any expected count fell below 5.

The primary analysis compared outcomes by prehospital classification within each ED-arrival stratum separately. Event proportions carry Wilson 95% confidence intervals, risk differences carry Newcombe intervals, and relative risks carry Katz intervals. Adjusted odds ratios come from logistic models containing age, sex, and comorbidity burden, with profile-likelihood confidence intervals. Whether the prehospital association differed between strata was tested formally. A single model was fitted on the whole cohort containing the ED classification, the prehospital classification, their interaction, and the same covariates, then compared against the main-effects model by likelihood-ratio test. Because five secondary outcomes were examined in each stratum, their *p* values were also adjusted for multiplicity by the Holm procedure, once across the five secondary outcomes within each stratum and once across all twelve within-stratum contrasts.

The incremental value of the prehospital value over the arrival value was assessed by nested logistic models for 30-day mortality: the arrival classification alone, then the arrival classification plus the prehospital classification. The two were compared by likelihood-ratio test, by the Akaike information criterion, and by areas under the receiver operating characteristic curve with DeLong intervals and the paired DeLong test. All discrimination comparisons were computed from the fitted probabilities of logistic models, so an ordinal score was never compared against a dichotomised one. Continuous and categorical net reclassification improvement and integrated discrimination improvement were computed for adding the prehospital value on top of the arrival value, following the Pencina definitions with 1000-resample bootstrap percentile intervals. Categorical cut-offs were set at predicted risks of 10% and 30%, with a second pair at 20% and 40%. Both choices are arbitrary. No accepted action threshold exists. Optimism-corrected discrimination, calibration, and Brier scores come from 1000 bootstrap resamples, and net benefit was computed across threshold probabilities from 1% to 60%. The whole comparison was repeated with both scores entered as ordinal 0 to 3 terms. A comparison against the arrival classification alone shows what the field value adds to that score. It does not show what the field value adds to the information a physician has at presentation. The nested comparison was therefore repeated against an extended reference model containing the arrival classification together with age, sex, comorbidity burden, and the lactate, oxygen saturation, heart rate, and temperature recorded at ED presentation. The same extended covariate set was then used to re-estimate the within-stratum odds ratios and the interaction term.

Agreement between the two measurements was quantified by percent agreement, Cohen kappa on the dichotomised classification, linearly weighted kappa on the ordinal scale, and the McNemar test for asymmetry in threshold crossing. Kappa was interpreted against the Landis and Koch benchmarks [29].

Precision is reported rather than retrospective power. For each within-stratum contrast, we give two quantities: the half-width of the observed 95% interval on the risk difference, and the smallest risk difference that would have been detectable at 80% power and two-sided alpha of 0.05 at the observed cell sizes and baseline risk.

Three sensitivity analyses were pre-specified. They were complete-case versus multiply imputed estimation, an ordinal alternative to the dichotomised threshold, and restriction to Sepsis-3-positive patients. A planned analysis excluding patients with treatment-limitation orders could not be performed, because that variable is not recorded.

The stratification described here was defined after cohort assembly. All stratified analyses are therefore exploratory and are labelled as such throughout. Every quantity reported here was recomputed from the source data by a second, independently written script. The two computations agreed on all 37 headline quantities examined in the primary analysis and on all 28 quantities of the analyses added at revision.

The possible effect of cohort selection on the primary comparison was examined in three ways rather than discussed. First, the primary contrast was repeated within strata of emergency department disposition, separating patients discharged from the department from those admitted or dying there, because selection on the availability of an in-hospital record would be expected to act through disposition. Second, the observed risk ratios were converted into selection bias bounds using the summary measures of Smith and VanderWeele, which give the minimum strength of the relationships between the unmeasured factors responsible for selection, the exposure, and the outcome that would be needed to move the risk ratio to 1; both the assumption-free bound and the bound that applies when selection is associated with increased outcome risk in both exposure groups are reported [30]. Third, a tipping-point analysis returned the 1481 excluded patients to the cohort across a grid of 27 scenarios per stratum, varying their distribution between the two triage strata, the prevalence of a positive prehospital classification among them, and their mortality level, and solving in each scenario for the risk ratio among the excluded patients that would bring the pooled within-stratum risk ratio to 1. A non-parametric envelope for the 266 transferred patients was computed in the same framework.

Ethical approval was granted by the Non-Interventional Clinical Research Ethics Committee of Bolu Abant İzzet Baysal University Faculty of Medicine (protocol code 2025/165, 22 April 2025), with a waiver of individual informed consent.

## 3. Results

### 3.1. Cohort

Of 17,786 adults delivered to the ED by 112 EMS during the three-year period, 2558 met the eligibility criteria for suspected infection, and 1077 (42.1%) were analysed (Figure 1). Median age was 77 years (IQR 68 to 85), 578 patients (53.7%) were male, and 896 (83.2%) met Sepsis-3 criteria. Median lactate was 1.70 mmol/L (IQR 1.15 to 2.56) and median SOFA score was 3 (IQR 2 to 6).

Patient-level exclusion during cohort assembly and variable-level missingness within the analysed cohort are different quantities, and we report them separately. At the patient level, 1481 of 2558 eligible patients (57.9%) were excluded before analysis, 1175 of them because the in-hospital record was incomplete (Figure 1). Nothing is known about these patients beyond the reason for their exclusion. Within the 1077 analysed patients, variable-level missingness was small. Of the 48 variables entering any analysis, 44 were complete in all 1077 patients. The four exceptions were one text-coded hypertension entry, the comorbidity count derived from it, two physiologically impossible Glasgow Coma Scale values that were set to missing where the scale entered an analysis as a continuous variable, and three missing C-reactive protein values that were not used in any model. Both qSOFA scores were complete, as were all three prehospital fields, every outcome, age, sex, and disposition. Adjusted models in the triage-negative stratum therefore fit on 684 patients rather than 685.

Prehospital qSOFA was 2 or more in 325 patients (30.2%) and ED-arrival qSOFA was 2 or more in 392 (36.4%). Within 30 days, 298 patients (27.7%) died. Intubation occurred in 129 (12.0%), ICU admission in 484 (44.9%), and the composite organ support outcome in 511 (47.4%). Median total length of stay was 5 days (IQR 0 to 13). Characteristics of the four ED-first cells are given in Table 1. Age rose modestly across the cells: median 75, 77, 79, and 80 years (*p* < 0.001). Comorbidity burden did not differ (*p* = 0.096).

### 3.2. The Two Measurements Frequently Disagree

The prehospital and arrival scores gave the same ordinal value in 454 of 1077 patients (42.2%). The linearly weighted kappa was 0.292 (95% CI 0.249 to 0.335). The ordinal score rose between the two measurements in 333 patients (30.9%) and fell in 290 (26.9%). Dichotomised at the conventional threshold, the two classifications agreed in 69.5% of patients, with a Cohen kappa of 0.315 (95% CI 0.256 to 0.374), which is fair agreement on the Landis and Koch scale [29].

**Figure 2:**
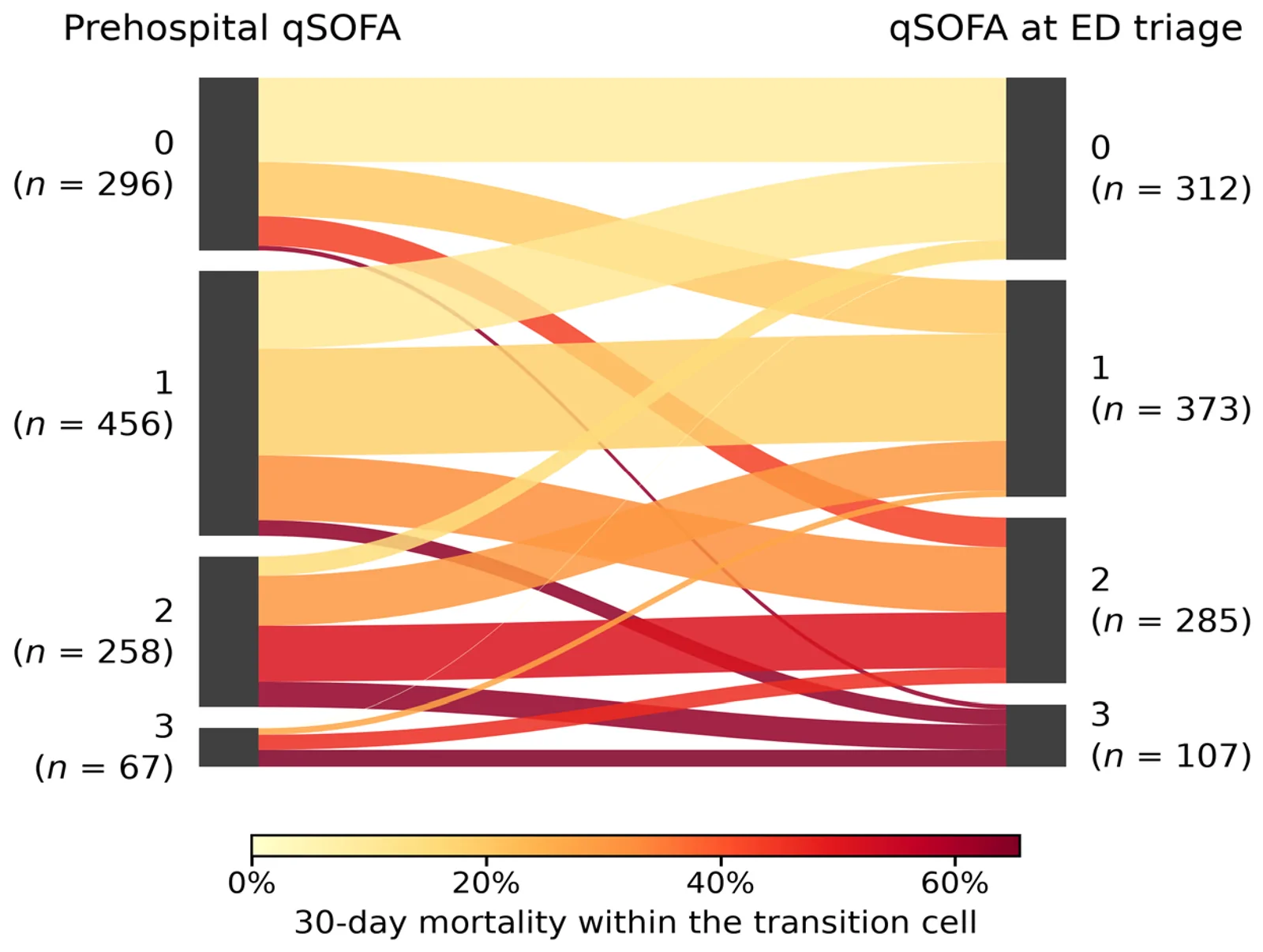
Transitions between the prehospital and emergency department arrival qSOFA values. Ribbon width is proportional to the number of patients moving between the two ordinal values, and ribbon colour to the 30-day mortality observed within that transition cell. The two measurements gave the same ordinal value in 454 of 1077 patients (42.2%). ED, emergency department; qSOFA, quick Sequential Organ Failure Assessment.

Disagreement was not symmetrical. Of the 1077 patients, 198 were prehospital-negative and arrival-positive, and 131 were prehospital-positive and arrival-negative (McNemar *p* = 0.00027). Table 2 gives the agreement statistics and Figure 2 shows the full transition pattern across the ordinal scale, coloured by the mortality observed within each transition cell. Two patients arriving at the same triage classification can therefore have travelled there from opposite directions. Roughly three in ten do.

### 3.3. Mortality by Prehospital Classification Within Each Triage Stratum

Among the 685 patients who were qSOFA-negative at triage, 30-day mortality was 13.9% (95% CI 11.3 to 17.0) when the field score had also been below 2, and 26.0% (19.2 to 34.1) when the field score had been 2 or more. That is a risk difference of 12.1 percentage points (95% CI 4.3 to 21.0), a relative risk of 1.87 (1.31 to 2.67), and an adjusted odds ratio of 2.06 (1.28 to 3.25; Fisher’s exact *p* = 0.001).

Among the 392 patients who were qSOFA-positive at triage, mortality was 40.4% (33.8 to 47.4) with a field score below 2 and 55.2% (48.1 to 62.0) with a field score of 2 or more. That is a risk difference of 14.8 percentage points (4.5 to 24.6), a relative risk of 1.37 (1.10 to 1.69), and an adjusted odds ratio of 1.84 (1.23 to 2.76; *p* = 0.005).

**Figure 3:**
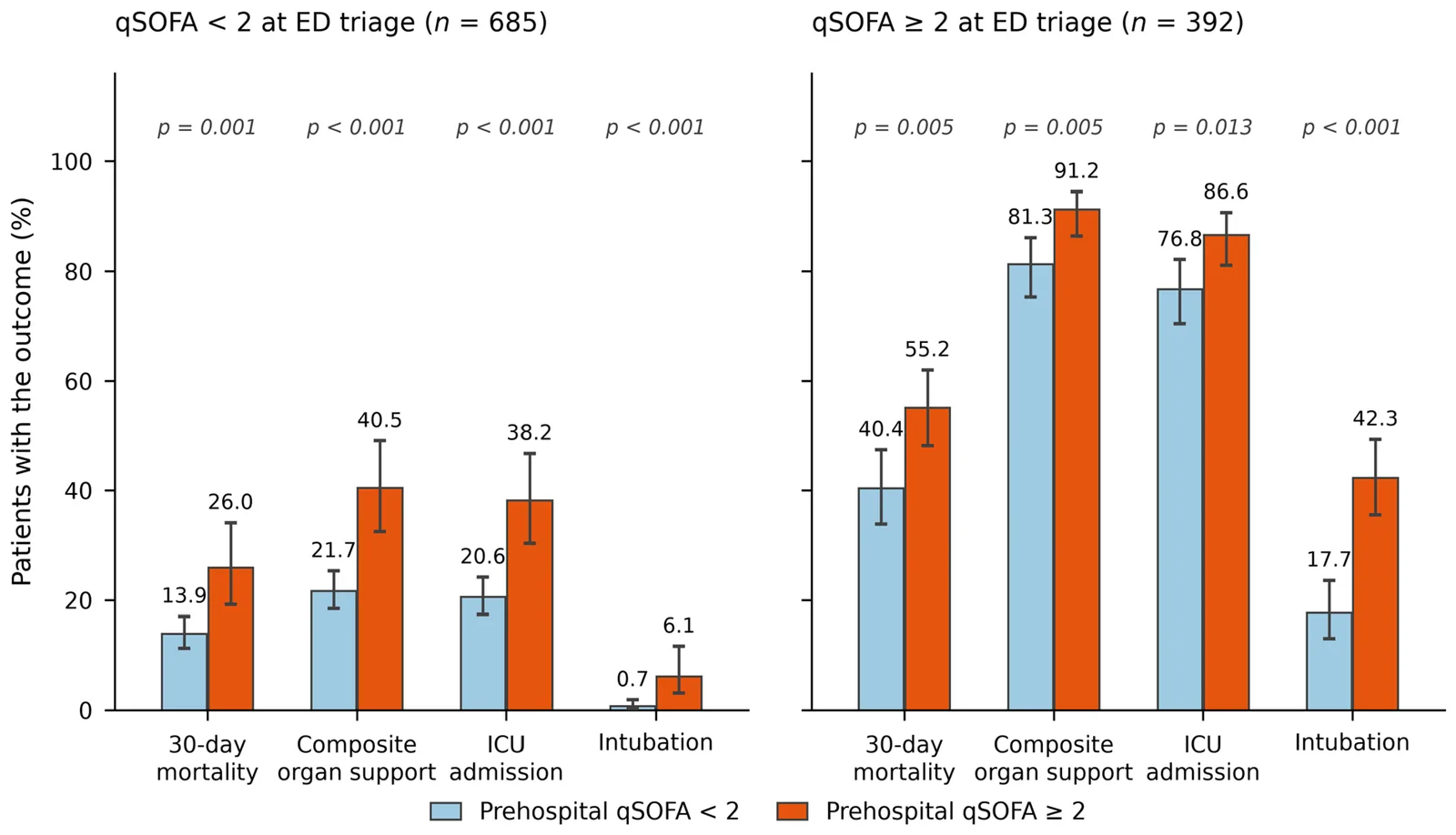
Outcomes by prehospital qSOFA classification within each emergency department triage stratum. Bars give observed event proportions with Wilson 95% confidence intervals. The left panel contains the 685 patients whose qSOFA was below 2 at emergency department triage and the right panel the 392 patients whose qSOFA was 2 or more. Composite organ support is any of intubation, vasopressor use, or intensive care unit admission. *p* values are from Fisher’s exact tests. ED, emergency department; ICU, intensive care unit; qSOFA, quick Sequential Organ Failure Assessment.

Every secondary outcome followed the same ordering in both strata (Table 3; Figure 3). In the triage-negative stratum, composite organ support rose from 21.7% to 40.5% and ICU admission from 20.6% to 38.2%. In the triage-positive stratum, the same outcomes rose from 81.3% to 91.2% and from 76.8% to 86.6%. Intubation was the most sharply separated outcome in relative terms, rising from 0.7% to 6.1% in the triage-negative stratum and from 17.7% to 42.3% in the triage-positive stratum. After Holm adjustment across the five secondary outcomes within each stratum, every secondary contrast remained below 0.05; across all twelve within-stratum contrasts the largest Holm-adjusted *p* value was 0.026 (Supplementary Table S7). Under the more conservative Bonferroni correction across twelve tests, five contrasts exceeded 0.05, among them 30-day mortality in the triage-positive stratum (*p* = 0.055).

The adjusted odds ratios in Table 3 control for age, sex, and comorbidity burden only. The extended covariate set adds lactate, oxygen saturation, heart rate, and temperature at presentation. With it, the adjusted odds ratio for 30-day death associated with a prehospital score of 2 or more was 1.76 (95% CI 1.07 to 2.83) in the triage-negative stratum and 1.65 (1.08 to 2.54) in the triage-positive stratum. For the secondary outcomes, the extended model estimates kept the same direction in every case; the intervals for ICU admission and composite organ support in the triage-positive stratum crossed 1 (Supplementary Table S8).

The achieved precision was adequate for the primary contrasts and no better than that. The half-width of the 95% interval on the mortality risk difference was 8.4 percentage points in the triage-negative stratum and 10.0 in the triage-positive stratum, and the smallest risk difference detectable at 80% power was 10.3 and 14.1 percentage points respectively. Both observed differences exceeded those thresholds. Smaller effects on the secondary outcomes would have escaped detection.

### 3.4. No Evidence of Heterogeneity Between Strata

Because the two stratum-specific odds ratios were similar, we tested whether they differ. In the full interaction model, the triage classification carried an adjusted odds ratio of 4.11 (95% CI 2.82 to 5.99) and the prehospital classification 2.11 (1.32 to 3.33). The interaction term was 0.850 (0.462 to 1.576), with a likelihood-ratio chi-square of 0.271 on 1 df (*p* = 0.602). The same interaction term was non-significant for every secondary outcome (Table 4), and it was 0.92 (0.49 to 1.74; *p* = 0.79) with the extended covariate set.

These data give no evidence that the prehospital association differs between triage strata. They do not show that it is the same. The interval on the interaction term spans a 3.4-fold range, from 0.46 to 1.58, and is compatible with an association that is substantially stronger in either stratum. The two stratum-specific estimates should therefore be read as separate estimates that happen to be similar, and the absence of detectable heterogeneity should not be read as equivalence.

### 3.5. Incremental Value over the Arrival Score

**Figure 4:**
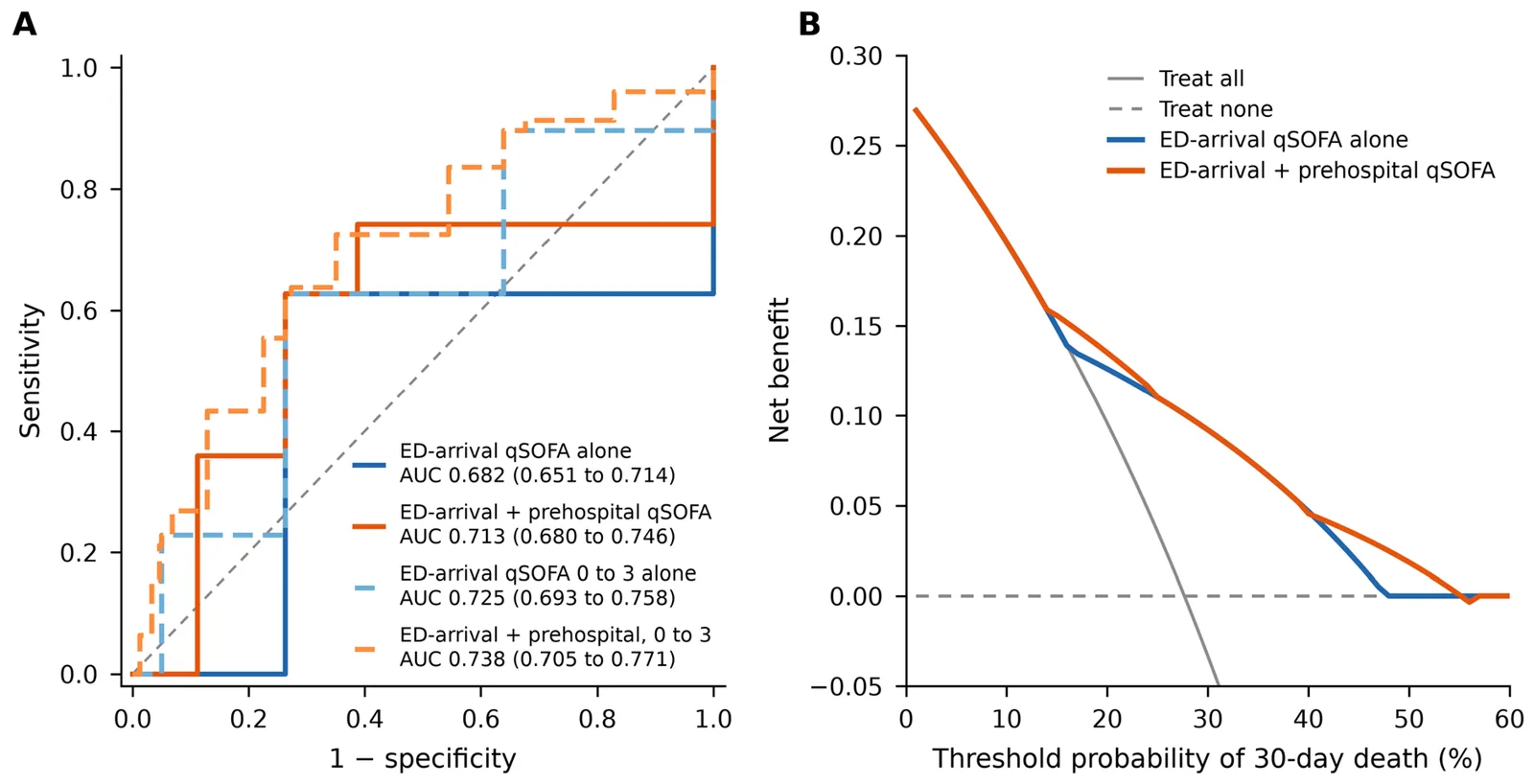
Discrimination and net benefit of adding the prehospital qSOFA classification to the arrival classification. (**A**) Receiver operating characteristic curves derived from the fitted probabilities of nested logistic models for 30-day mortality, with DeLong 95% confidence intervals for each area under the curve. Solid lines are the dichotomised models and dashed lines the ordinal sensitivity analysis. (**B**) Decision curve analysis for the two dichotomised models against treat-all and treat-none strategies. AUC, area under the receiver operating characteristic curve; ED, emergency department; qSOFA, quick Sequential Organ Failure Assessment.

The nested model comparison is given in Table 5. Adding the prehospital classification to the arrival classification alone improved fit: likelihood-ratio chi-square 18.54 on 1 df (*p* < 0.001), with the Akaike information criterion falling from 1153.6 to 1137.0. Discrimination for 30-day mortality rose from 0.682 (95% CI 0.651 to 0.714) to 0.713 (0.680 to 0.746), a difference of 0.031 by the paired DeLong test (*p* < 0.001). Optimism-corrected values were 0.683 and 0.714 (Figure 4A). In the two-variable model, the prehospital classification retained an odds ratio of 1.960 (1.445 to 2.655). The continuous net reclassification improvement was 0.474 (0.342 to 0.599) and the integrated discrimination improvement 0.0173 (0.0042 to 0.0359). The categorical index at fixed cut-offs was zero or negative, for the structural reason set out in Supplementary Table S10. We do not rely on it. Decision curve analysis showed a small increment in net benefit for the two-variable model across threshold probabilities from roughly 14% to 56%, with the two curves converging outside that range (Figure 4B).

That comparison establishes what the field value adds to the arrival classification alone. It does not establish what it adds to the information a physician already has at presentation. The extended reference model contained the arrival classification with age, sex, comorbidity burden, lactate, oxygen saturation, heart rate, and temperature, and by itself reached an AUC of 0.751 (0.719 to 0.783). Adding the prehospital classification to it remained statistically informative (likelihood-ratio chi-square 10.81 on 1 df, *p* = 0.001; adjusted odds ratio 1.71, 1.24 to 2.34). The AUC rose by only 0.007, to 0.758 (0.726 to 0.789; DeLong *p* = 0.23). The integrated discrimination improvement was 0.010 (0.001 to 0.024), and the optimism-corrected AUCs were 0.743 and 0.749. With both scores entered as ordinal terms, the likelihood-ratio *p* was 0.071 and the AUC difference 0.003 (*p* = 0.32). The incremental value of the field score is therefore measurable relative to the arrival score alone and small relative to routine ED information (Supplementary Table S9).

The ordinal sensitivity analysis of the two-score comparison reproduced the gain on a different scale. With both scores entered as 0 to 3 terms, adding the prehospital score gave a likelihood-ratio chi-square of 5.35 on 1 df (*p* = 0.021). The AUC rose from 0.725 (0.693 to 0.758) to 0.738 (0.705 to 0.771), with a paired DeLong *p* of 0.011, and the prehospital score carried an odds ratio of 1.231 (1.032 to 1.468) per point.

### 3.6. Secondary and Sensitivity Analyses

Multiple imputation reproduced the primary estimates. The pooled adjusted odds ratio was identical to the complete-case value in the triage-positive stratum (1.839), and differed in the third decimal in the triage-negative stratum (2.057 versus 2.056). Restriction to the 896 Sepsis-3-positive patients preserved the pattern. Prehospital qSOFA of 2 or more had a sensitivity of 34.4% (95% CI 31.3 to 37.5) and a specificity of 90.6% (85.5 to 94.1) for Sepsis-3. The arrival score reached 43.4% (40.2 to 46.7) and 98.3% (95.2 to 99.4). A positive predictive value of 94.8% reflects the 83.2% prevalence in this cohort, and falls to 28.9% at a prevalence of 10% (Supplementary Table S4).

The overlap between the qSOFA items and the SOFA domains defining the reference standard was quantified rather than only acknowledged. Removing the neurological sub-score from the reconstructed reference standard lowered specificity by 4.5 percentage points and changed sensitivity by 0.2; removing all three overlapping domains lowered specificity by 15.9 points and changed sensitivity by 0.4. The insensitivity of the score is therefore not an artefact of incorporation, whereas its apparently high specificity partly is. Supplementary Tables S1–S10 carry the remaining detail. They give the missingness audit, the four-group trajectory view, the component-level analysis, the diagnostic accuracy analysis, the sensitivity analyses, a comparison against previously published prehospital qSOFA studies, the multiplicity-adjusted *p* values, the extended covariate stratum estimates, the extended reference model, and the reclassification indices.

### 3.7. Cohort Selection and What Would Be Needed to Explain the Association

Selection acted at the level of the record, so its effect cannot be measured directly. Three analyses bound it. The first tests the mechanism itself. If requiring an in-hospital record had selected sicker, investigated patients, the association should be weaker or absent among patients who were sent home from the emergency department. It was not. Of the 1077 analysed patients, 278 (25.8%) were discharged from the department, and their 30-day mortality was 10.1%. Within the triage-negative stratum, 30-day mortality among these discharged patients was 7.6% (17 of 225) when the field score had been below 2 and 21.3% (10 of 47) when it had been 2 or more, a risk ratio of 2.82 (95% CI 1.38 to 5.76) and an adjusted odds ratio of 2.90 (1.17 to 6.86). Among admitted patients, the same contrast gave 18.2% versus 28.6% in the triage-negative stratum (risk ratio 1.57, 1.04 to 2.36) and 40.3% versus 56.3% in the triage-positive stratum (1.40, 1.13 to 1.73). The interaction between the prehospital classification and disposition was not significant (odds ratio 1.31, 0.52 to 3.22; *p* = 0.56). The association is therefore present in the subgroup that selection on record availability would be most likely to deplete (Supplementary Table S12).

The second analysis asks how strong selection would have to be. On the assumption-free bound, each of the four parameters linking the unmeasured selection factors to exposure and outcome would have to reach 2.07 in the triage-negative stratum and 1.61 in the triage-positive stratum to move the observed risk ratios to 1; taking the lower confidence limits instead, 1.55 and 1.28. If selection is assumed to be associated with increased mortality risk in both prehospital groups, which is the mechanism proposed for this cohort, the corresponding values are 3.14 and 2.07 (1.94 and 1.45 at the lower confidence limits) [30].

The third analysis returns the excluded patients to the cohort. If they had shown the same association as the analysed patients, the pooled risk ratios would be unchanged at 1.87 and 1.37 in the base case, and would lie between 1.57 and 2.42 in the triage-negative stratum and between 0.96 and 1.95 in the triage-positive stratum across the full grid of assumptions. To reach 1, the 1481 excluded patients would have to show a reversed association. In the base-case scenario, the required risk ratio among them is 0.37 in the triage-negative stratum and 0.73 in the triage-positive stratum, that is, field-positive patients dying less often than field-negative ones. Across the 27 scenarios, no value of the excluded-group risk ratio could bring the triage-negative estimate to 1 in 17 scenarios, and in the remaining 10, the required value ranged from 0.06 to 0.69, always a reversal. The triage-positive stratum was less robust: 10 scenarios could not be nullified, 13 required a reversed association, and in 4, the estimate reached 1 with a positive association among the excluded patients (1.03 to 1.49). All four of those scenarios combine a prehospital-positive prevalence half again as high among the excluded patients with a mortality level a quarter to a half of that observed. For the 266 transferred patients considered alone, the non-parametric envelope is wide and includes 1 (0.67 to 3.64 and 0.85 to 1.98), as it must be when 266 outcomes are unknown (Supplementary Table S13).

## 4. Discussion

In 1077 ambulance-transported adults with suspected infection, the prehospital qSOFA classification was associated with 30-day mortality within each ED triage classification. Two patients arriving with the same triage score, both negative, died at 13.9% and 26.0% depending on what the crew had recorded at the scene. Two patients arriving both triage-positive died at 40.4% and 55.2%. The association was present for every outcome we examined and we found no evidence that it differed between the two triage strata. Adding the field value to the arrival value alone improved discrimination for death by 0.031. Adding it to a model that already held the age, comorbidity, lactate, oxygen saturation, heart rate, and temperature recorded at presentation left the association intact but improved discrimination by 0.007, a change that was not distinguishable from zero.

Both results matter. They have to be read together. The field score contains prognostic information that the arrival score does not, and most of that information is also carried by measurements the physician already has. What remains specific to the field score is an independent association with death of modest size, in a cohort selected in ways we describe below.

### 4.1. Relation to Previous Work

Several papers in this journal approach the question of what the field score adds once the patient has arrived, and none of them asks the question we ask. Dankert and colleagues linked prehospital qSOFA parameters to the timing of targeted sepsis therapy in 702 single-centre patients, an endpoint about process rather than outcome [31]. Kornfehl and colleagues measured how accurately EMS crews detect sepsis and how three field scores perform as detection instruments, over one month and twelve sepsis events [17]. Devia-Jaramillo and colleagues compared qSOFA against the National Early Warning Score and the International Early Warning Score at triage, establishing the arrival-side ranking without a field measurement [32]. Martin-Rodriguez and colleagues showed that adding point-of-care lactate to a prehospital score improves stratification, that is, information added to the field value [33]. We ask the reverse question: what the field value adds to the arrival value. The closest published work is a cohort of 86,069 EMS-transported non-trauma patients classified as low risk at ED triage. There, in-hospital mortality rose across prehospital NEWS categories from 1.2% to 6.8%, and the authors concluded that normalisation of physiology before arrival does not indicate resolution of risk [25]. That study used a different score, a much larger cohort, and only the low-risk triage stratum. We observe the same pattern with qSOFA, in both triage strata, with a formal test for heterogeneity between them.

For qSOFA itself, the nearest comparison is a Japanese cohort of 2407 ambulance-transported adults with suspected infection, in which prehospital and ED qSOFA had indistinguishable c-statistics for sepsis, yet a combined field-and-department score identified 44 of 369 sepsis cases (12%) that the ED score alone would have missed [26]. Those authors framed the question as case identification; we frame it as a prognostic association with death and organ support, stratified by the arrival classification. A prehospital-to-hospital qSOFA trajectory has also predicted mortality in 90,974 trauma patients [23], and tracking NEWS2 from scene to triage improved discrimination in 4943 ambulance patients [24]. Inside the hospital, repeated qSOFA measurement outperforms a single reading [20,21], although serial measurement at 30 min intervals added little in a smaller series [22], and a prospective prehospital cohort found qSOFA discriminated septic shock better than NEWS2 [34]. Our contribution is to move that logic one interval earlier and to show how much of it survives adjustment for what the receiving physician can see.

### 4.2. What the Field Score Is for

The 2026 Surviving Sepsis Campaign guideline recommends NEWS, NEWS2, MEWS, or SIRS over qSOFA as a single in-hospital screening tool, and records qSOFA as having the lowest prehospital sepsis sensitivity among the instruments reviewed, 23.1% [19]. We do not contest that. We measured a sensitivity of 34.4% for Sepsis-3, which belongs in the same unimpressive range as the published estimates [10,11,12], and we would not propose qSOFA as a field screening test on this evidence.

Our observation concerns a different property of the same number. Screening asks whether a positive result should trigger an action at the moment of measurement; our question asks whether an already-recorded value changes the interpretation of a later one. The guideline addresses only the first. On the second, a field qSOFA of 2 or more was associated with roughly twice the adjusted odds of death whatever the triage score turned out to be. Once lactate and the other arrival measurements were in the model, the ratio was 1.7. This bears on EMS documentation rather than EMS action: the recorded value retains some prognostic content for a clinician the crew will never meet.

That information is also non-recoverable once the handover is over. In 200 advanced-life-support transports, only 30% of third blood pressure readings on the handover form matched the definitive care report [35]. Among 3528 adults brought to a trauma centre by paramedics, each of four vital signs was missing in about 20%, and mortality was higher in those with incomplete data [36]. The same constraint is documented in the Turkish system. In a prospective cardiac arrest series from a tertiary centre, prehospital data had to be assembled from ambulance charts, relatives and attending providers, and remained too incomplete to analyse because Utstein-style reporting was not in routine use [37]. Field physiology that is not written down at the time cannot be reconstructed later.

### 4.3. Clinical Implications

The implications for the physician at triage are modest. They should be stated as such. In our cohort, a reassuring triage qSOFA identified a group whose 30-day mortality was 13.9%, and within it, the 131 patients who had been field-positive died at 26.0%; on the triage sheet, the two subgroups look identical. A field-positive score that has normalised on arrival was not associated with a resolved risk, which matches the conclusion drawn from prehospital NEWS in a far larger cohort [25]. At the same time the two-variable model reached an AUC of 0.713 and the extended model 0.758, and neither would justify changing an individual disposition decision on its own. Discrimination in that band is usual for physiology-based prediction in ED populations: a single laboratory ratio reached 0.718 for 30-day mortality in another Turkish ED cohort [38], and physiological and laboratory values at presentation carried the prognostic signal in a series of necrotizing fasciitis from our group [39].

We therefore do not propose a new score, a decision rule, or a change in disposition practice. The data support something narrower: that prehospital physiological information should be preserved at handover and considered alongside the arrival assessment, and that its prognostic value deserves prospective evaluation in a cohort assembled without the selection described below. The incremental logic is one we have applied before: adding a field-measured variable to an existing prehospital rule changed that rule’s operating characteristics without replacing it [40]. Whether reading the field score changes any decision, and whether that improves outcome, are questions this design cannot answer.

### 4.4. Why the Two Measurements Differ

We cannot say why the scores changed. Three explanations remain open. Genuine physiological change, measurement variability in either setting, and response to prehospital treatment are all possible; prehospital treatments and the interval between the measurements are not recorded, so they cannot be separated. Some measurement variability is certain, because prehospital vital signs are collected in a moving vehicle by a crew managing a patient and both scores depend on a Glasgow Coma Scale assessment, the least reproducible of the three items.

There is also a structural point. The ED classification is measured after the prehospital one and may itself reflect underlying severity, spontaneous change and treatment en route. Stratifying on it produces clinically intuitive groups, but it conditions the analysis on a post-baseline variable. We therefore describe a prognostic association between two serial measurements and an outcome and make no mechanistic or causal claim about the change between them. The prognostic reading holds whichever mechanism produced the difference: the field-positive, triage-negative patient may have improved, been treated, or been measured differently, and that patient’s 30-day mortality was 26.0% either way.

### 4.5. Limitations

The design is retrospective and single-centre, and the cohort is highly selected. Only 1077 of 2558 eligible patients (42.1%) entered the analysis. The largest group of exclusions, 1175 patients, arose because the hospital episode record could not be retrieved, which removed the arrival measurement and the outcome together; 266 were transferred before an outcome could be ascertained and 40 left before evaluation. No data on these patients were retained and the approval covers the existing de-identified dataset only, so we cannot compare included with excluded patients, and the analysis cannot be reconstructed on a wider cohort. The direction of the resulting bias can be reasoned about: patients whose record was not retrievable are likely to include a disproportionate share of brief, undocumented attendances, so the analysed cohort probably over-represents patients who were investigated and admitted, which would inflate the observed mortality and the Sepsis-3 prevalence (83.2%) and shrink the triage-negative stratum. The quantitative analyses in Section 3.7 bound what this could do to the central estimate rather than leaving it to argument. Their answer is not uniform: the triage-negative association survives every plausible selection scenario we could construct, whereas the triage-positive association can be moved to 1 by a specific adverse combination, and it should be read with that in mind. Every proportion in this paper, including the stratum sizes, should be read as belonging to a selected cohort.

Eligibility and the non-infectious exclusions were adjudicated retrospectively by a single investigator who was not blinded to outcome, and no second assessor or agreement statistic is available. The eligibility criteria themselves include hypotension, tachypnea, and altered mental status, which are the qSOFA items, so some patients entered the cohort because of the physiology under study. Patients who had received prehospital advanced life support were excluded, which removes some of the sickest patients from both strata, and the number removed on that ground was not recorded.

The stratification was defined after the cohort had been assembled, and six outcomes were examined in each stratum. All stratified analyses are therefore exploratory. Holm adjustment left every secondary contrast below 0.05, but under a Bonferroni correction across twelve tests five contrasts did not, including 30-day mortality in the triage-positive stratum. The consistent direction of the findings and their agreement with an independent cohort using a different score [25] make chance an unlikely explanation for the pattern as a whole, but they do not substitute for confirmation in an independent cohort.

Adjustment was limited. The primary models contained age, sex, and a count of nine comorbidities; the extended models added lactate, oxygen saturation, heart rate, and temperature at presentation. Frailty, the source of infection, prehospital treatment, the transport interval and treatment-limitation status were not recorded and could not be adjusted for. In a cohort with a median age of 77 years, these omissions matter. Frailty in particular improves risk prediction over qSOFA and other physiological scores in older ED patients [41], and some deaths in the highest-risk cell may reflect decisions not to escalate care rather than failure of escalation. The interaction test had limited precision: its confidence interval spans a 3.4-fold range, so a non-significant result excludes only large differences between strata.

The interval between the two measurements is not recorded. We therefore cannot say over how many minutes the observed changes occurred, nor separate transport time from time to first ED assessment. The manuscript can claim only that the prehospital value carries information measured earlier, not that it carries information about a rate of change. Prehospital respiratory rate and consciousness exist in the source data only as the dichotomous criteria, so no continuous field values exist and no model of who converts could be built. Prehospital treatments, oxygen saturation, temperature, and lactate are not recorded either. Date of death is not recorded, only 30-day status, so no time-to-event analysis was possible.

The Sepsis-3 secondary analysis retains incorporation bias, because the qSOFA items overlap the SOFA domains that define the reference standard. We bounded the direction and size of that overlap rather than only naming it, and the effect fell almost entirely on specificity. Finally, the arrival measurement is temporally closer to the outcome than the field measurement, which favours it in any discrimination comparison. That is intrinsic to the question rather than a correctable feature of the design. It is also the reason the analysis is framed as the incremental value of the earlier measurement over the later one, not as a contest between them.

## 5. Conclusions

In ambulance-transported adults with suspected infection, the prehospital qSOFA classification recorded by the ambulance crew was associated with 30-day mortality within each emergency department triage classification. A field score of 2 or more marked higher risk even in patients who were qSOFA-negative at triage, and the same pattern appeared for intubation, vasopressor use, intensive care admission, and composite organ support. There was no evidence that the association differed between the two strata. Added to the arrival score, the field value improved discrimination. Added to routinely available arrival data, it remained associated with death but changed discrimination very little.

These results are exploratory, come from a single centre, and rest on a cohort that retained 42% of eligible patients. Quantitative bias analysis indicates that the triage-negative association would survive any selection short of a reversed association among the excluded patients, whereas the triage-positive association could be nullified in most of the scenarios examined, in four of them by a plausible positive association. The results support recording prehospital physiology and passing it on at handover. They do not support changing disposition decisions, and they justify prospective validation.

## Figures and Tables

**Table 1:** Characteristics of the cohort by emergency department triage stratum and prehospital qSOFA classification.

Characteristic	qSOFA < 2 at Triage, Prehospital < 2 (*n* = 554)	qSOFA < 2 at Triage, Prehospital ≥ 2 (*n* = 131)	qSOFA ≥ 2 at Triage, Prehospital < 2 (*n* = 198)	qSOFA ≥ 2 at Triage, Prehospital ≥ 2 (*n* = 194)	*p* Value *
Demographics and comorbidity					
Age, years, median (IQR)	75 (66–84)	77 (69–84)	79 (71–85)	80 (72–87)	<0.001
Male sex, *n* (%)	307 (55.4)	62 (47.3)	100 (50.5)	109 (56.2)	0.252
Comorbidity burden (0 to 9), median (IQR)	2 (1–3)	2 (1–3)	3 (1–3)	2 (1–3)	0.096
Hypertension, *n* (%)	335 (60.4)	77 (58.8)	131 (66.2)	122 (62.9)	0.448
Diabetes mellitus, *n* (%)	205 (37.0)	50 (38.2)	72 (36.4)	76 (39.2)	0.936
Coronary artery disease, *n* (%)	180 (32.5)	42 (32.1)	68 (34.3)	67 (34.5)	0.925
Chronic obstructive pulmonary disease, *n* (%)	112 (20.2)	32 (24.4)	53 (26.8)	55 (28.4)	0.068
Heart failure, *n* (%)	89 (16.1)	27 (20.6)	44 (22.2)	46 (23.7)	0.060
Chronic kidney disease, *n* (%)	83 (15.0)	20 (15.3)	28 (14.1)	21 (10.8)	0.528
Malignancy, *n* (%)	118 (21.3)	25 (19.1)	45 (22.7)	37 (19.1)	0.775
Liver disease, *n* (%)	9 (1.6)	2 (1.5)	4 (2.0)	3 (1.5)	0.982
Cerebrovascular disease, *n* (%)	105 (19.0)	32 (24.4)	46 (23.2)	42 (21.6)	0.396
Prehospital measurements					
Prehospital respiratory criterion met, *n* (%)	163 (29.4)	90 (68.7)	67 (33.8)	160 (82.5)	<0.001
Prehospital altered mentation criterion met, *n* (%)	41 (7.4)	78 (59.5)	20 (10.1)	129 (66.5)	<0.001
Prehospital systolic blood pressure ≤100 mmHg, *n* (%)	113 (20.4)	106 (80.9)	52 (26.3)	154 (79.4)	<0.001
Prehospital systolic blood pressure, mmHg, median (IQR)	130 (110–140)	100 (90–100)	120 (100–140)	90 (80–100)	<0.001
Emergency department measurements at triage					
Triage respiratory criterion met, *n* (%)	139 (25.1)	31 (23.7)	163 (82.3)	180 (92.8)	<0.001
Triage altered mentation criterion met, *n* (%)	87 (15.7)	57 (43.5)	182 (91.9)	177 (91.2)	<0.001
Triage systolic blood pressure ≤100 mmHg, *n* (%)	50 (9.0)	9 (6.9)	86 (43.4)	103 (53.1)	<0.001
Respiratory rate at triage, breaths/min, median (IQR)	18 (16–22)	20 (18–20)	26 (24–30)	28 (24–34)	<0.001
Systolic blood pressure at triage, mmHg, median (IQR)	128 (113–145)	121 (110–138)	110 (94–133)	100 (85–122)	<0.001
Diastolic blood pressure at triage, mmHg, median (IQR)	70 (60–81)	70 (62–79)	62 (50–79)	60 (47–70)	<0.001
Mean arterial pressure at triage, mmHg, median (IQR)	90.5 (80.0–103.3)	88.7 (80.3–96.3)	79.7 (66.7–95.6)	71.3 (60.0–85.8)	<0.001
Heart rate at triage, beats/min, median (IQR)	99 (85–115)	97 (82–110)	104 (88–121)	105 (86–127)	<0.001
Glasgow Coma Scale score at triage, median (IQR)	15 (15–15)	15 (14–15)	14 (11–14)	11 (8–14)	<0.001
Peripheral oxygen saturation at triage, %, median (IQR)	92 (89–94)	90 (88–94)	87 (80–90)	84 (78–89)	<0.001
Temperature at triage, °C, median (IQR)	37.0 (36.5–37.6)	36.7 (36.5–37.2)	37.0 (36.5–37.9)	36.9 (36.5–37.5)	0.018
Lactate, mmol/L, median (IQR)	1.50 (1.08–2.11)	1.61 (1.25–2.60)	2.02 (1.27–3.02)	2.17 (1.50–3.78)	<0.001
SOFA score, median (IQR)	2 (1–4)	3 (2–5)	5 (4–8)	7 (5–9)	<0.001
Sepsis-3 criteria met, *n* (%)	392 (70.8)	115 (87.8)	196 (99.0)	193 (99.5)	<0.001

Patients are grouped first by their qSOFA classification at emergency department triage and then by their prehospital classification, both at the conventional threshold of 2 or more. * Kruskal–Wallis test across the four cells for continuous variables and Pearson chi-square or Fisher’s exact test for categorical variables. Continuous variables are given as medians with interquartile ranges because Shapiro–Wilk testing returned *p* < 0.05 within at least one cell for every variable listed. Comorbidity burden is the count of nine recorded conditions. IQR, interquartile range; qSOFA, quick Sequential Organ Failure Assessment; SOFA, Sequential Organ Failure Assessment.

**Table 2:** Agreement between the prehospital and emergency department arrival qSOFA measurement.

Agreement Measure	Value (95% CI)
Exact agreement on the ordinal 0 to 3 scale, %	42.2
Cohen unweighted kappa, ordinal scale	0.179 (0.137–0.220)
Linearly weighted kappa, ordinal scale	0.292 (0.249–0.335)
Exact agreement after dichotomising at 2 or more, %	69.5
Cohen kappa, dichotomised	0.315 (0.256–0.374)
Score increased between the two measurements, *n* (%)	333 (30.9)
Score unchanged, *n* (%)	454 (42.2)
Score decreased, *n* (%)	290 (26.9)
Prehospital < 2 and triage ≥ 2 (upward threshold crossing), *n*	198
Prehospital ≥ 2 and triage < 2 (downward threshold crossing), *n*	131
McNemar test of asymmetry in threshold crossing	*p* = 0.00027

Kappa values are interpreted against the Landis and Koch benchmarks. The McNemar test assesses whether upward and downward crossings of the threshold of 2 or more occur with equal frequency. CI, confidence interval; qSOFA, quick Sequential Organ Failure Assessment.

**Table 3:** Outcomes by prehospital qSOFA classification within each emergency department triage stratum.

Outcome	Prehospital qSOFA < 2, % (Events/*n*)	Prehospital qSOFA ≥ 2, % (Events/*n*)	Risk Difference, Percentage Points (95% CI)	Relative Risk (95% CI)	Adjusted Odds Ratio (95% CI) ‡	*p* Value §
Patients with qSOFA < 2 at emergency department triage (*n* = 685)						
30-day mortality	13.9 (77/554)	26.0 (34/131)	12.1 (4.3–21.0)	1.87 (1.31–2.67)	2.06 (1.28–3.25)	0.001
Composite organ support †	21.7 (120/554)	40.5 (53/131)	18.8 (9.6–28.3)	1.87 (1.44–2.43)	2.38 (1.58–3.57)	<0.001
Intensive care unit admission	20.6 (114/554)	38.2 (50/131)	17.6 (8.6–27.1)	1.85 (1.41–2.44)	2.29 (1.52–3.46)	<0.001
Intubation	0.7 (4/554)	6.1 (8/131)	5.4 (1.9–11.4)	8.46 (2.59–27.66)	8.20 (2.52–31.30)	<0.001
Vasopressor use in the emergency department	1.4 (8/554)	5.3 (7/131)	3.9 (0.6–9.7)	3.70 (1.37–10.02)	3.81 (1.30–10.91)	0.013
Vasopressor use on the ward	11.9 (66/554)	22.1 (29/131)	10.2 (3.0–18.9)	1.86 (1.25–2.75)	2.00 (1.21–3.24)	0.005
Patients with qSOFA ≥ 2 at emergency department triage (*n* = 392)						
30-day mortality	40.4 (80/198)	55.2 (107/194)	14.8 (4.5–24.6)	1.37 (1.10–1.69)	1.84 (1.23–2.76)	0.005
Composite organ support †	81.3 (161/198)	91.2 (177/194)	9.9 (2.7–17.1)	1.12 (1.04–1.22)	2.40 (1.31–4.55)	0.005
Intensive care unit admission	76.8 (152/198)	86.6 (168/194)	9.8 (1.8–17.7)	1.13 (1.03–1.24)	1.99 (1.18–3.43)	0.013
Intubation	17.7 (35/198)	42.3 (82/194)	24.6 (15.3–33.4)	2.39 (1.70–3.37)	3.38 (2.13–5.46)	<0.001
Vasopressor use in the emergency department	19.7 (39/198)	33.5 (65/194)	13.8 (4.7–22.6)	1.70 (1.21–2.40)	2.01 (1.27–3.22)	0.002
Vasopressor use on the ward	54.0 (107/198)	74.2 (144/194)	20.2 (10.4–29.5)	1.37 (1.18–1.60)	2.41 (1.58–3.72)	<0.001

† Composite organ support is any of intubation, vasopressor use, or intensive care unit admission. ‡ Adjusted for age, sex, and comorbidity burden by logistic regression with profile-likelihood confidence intervals; the triage-negative models fit on 684 of 685 patients because one comorbidity count is missing. § Fisher’s exact test, unadjusted for multiplicity; Holm-adjusted values are given in Supplementary Table S7. Risk differences carry Newcombe confidence intervals and relative risks carry Katz confidence intervals. This stratified analysis was defined after the cohort was assembled and is exploratory. CI, confidence interval; qSOFA, quick Sequential Organ Failure Assessment.

**Table 4:** Test for heterogeneity of the prehospital association between the two triage strata.

Model Term	Adjusted Odds Ratio (95% CI)	*p* Value
qSOFA ≥ 2 at emergency department triage	4.105 (2.822–5.989)	<0.001
Prehospital qSOFA ≥ 2	2.115 (1.323–3.335)	0.001
Triage × prehospital interaction	0.850 (0.462–1.576)	0.602
Age, per year	1.011 (0.999–1.023)	0.073
Male sex	0.990 (0.739–1.326)	0.945
Comorbidity burden, per condition	0.936 (0.843–1.038)	0.213
Likelihood-ratio test for the interaction term	χ^2^ = 0.271, 1 df	0.602
Prehospital association implied within the triage-negative stratum	2.115 (1.334–3.354)	
Prehospital association implied within the triage-positive stratum	1.797 (1.201–2.687)	
Interaction term for each secondary outcome		
Composite organ support	0.978 (0.474–2.073)	0.953
Intensive care unit admission	0.832 (0.428–1.638)	0.591
Intubation	0.401 (0.098–1.425)	0.160
Vasopressor use in the emergency department	0.542 (0.173–1.728)	0.293
Vasopressor use on the ward	1.190 (0.626–2.295)	0.598

A single logistic model was fitted on the whole cohort containing the triage classification, the prehospital classification, their interaction, age, sex, and comorbidity burden. The implied stratum-specific associations are the prehospital odds ratios that this model produces within each triage stratum. The likelihood-ratio test compares the full model against the main-effects model. A non-significant interaction term does not establish that the association is the same in both strata; see Section 3.4. CI, confidence interval; df, degrees of freedom; qSOFA, quick Sequential Organ Failure Assessment.

**Table 5:** Incremental value of the prehospital qSOFA classification for 30-day mortality over the emergency department arrival classification alone and over an extended clinical reference model.

Measure	Model 1: Triage qSOFA Alone	Model 2: Triage + Prehospital qSOFA	Difference or Test Result
Odds ratio for qSOFA ≥ 2 at triage (95% CI)	4.717 (3.559–6.281)	3.930 (2.928–5.292)	
Odds ratio for prehospital qSOFA ≥ 2 (95% CI)	not in model	1.960 (1.445–2.655)	
Akaike information criterion	1153.6	1137.0	−16.5
Likelihood-ratio test for adding the prehospital value			χ^2^ = 18.54, 1 df, *p* < 0.001
AUC (95% CI)	0.682 (0.651–0.714)	0.713 (0.680–0.746)	+0.031, DeLong *p* < 0.001
Optimism-corrected AUC	0.683	0.714	
Optimism-corrected Brier score	0.178	0.175	
Optimism-corrected calibration slope	1.010	1.002	
Integrated discrimination improvement (95% CI)			0.0173 (0.0042–0.0359)
Ordinal sensitivity analysis (both scores entered as 0 to 3)			
Odds ratio for the prehospital score, per point (95% CI)	not in model	1.231 (1.032–1.468)	
Likelihood-ratio test for adding the prehospital score			χ^2^ = 5.35, 1 df, *p* = 0.021
AUC (95% CI)	0.725 (0.693–0.758)	0.738 (0.705–0.771)	+0.013, DeLong *p* = 0.011
Integrated discrimination improvement (95% CI)			0.0048 (0.0000–0.0166)
Extended clinical reference model (triage qSOFA with age, sex, comorbidity burden, lactate, oxygen saturation, heart rate and temperature; *n* = 1076); Model 1 without and Model 2 with the prehospital classification			
Odds ratio for prehospital qSOFA ≥ 2 (95% CI)	not in model	1.709 (1.243–2.342)	
Odds ratio for qSOFA ≥ 2 at triage (95% CI)		3.079 (2.237–4.246)	
Akaike information criterion	1112.7	1103.9	−8.8
Likelihood-ratio test for adding the prehospital value			χ^2^ = 10.81, 1 df, *p* = 0.001
AUC (95% CI)	0.751 (0.719–0.783)	0.758 (0.726–0.789)	+0.007, DeLong *p* = 0.23
Optimism-corrected AUC	0.743	0.749	
Integrated discrimination improvement (95% CI)			0.010 (0.001–0.024)
Ordinal variant: likelihood-ratio test for adding the prehospital score			χ^2^ = 3.26, 1 df, *p* = 0.071
Ordinal variant: AUC (95% CI)	0.757 (0.725–0.789)	0.760 (0.728–0.791)	+0.003, DeLong *p* = 0.32

All models are logistic models for 30-day mortality, and all discrimination measures are computed from their fitted probabilities, so an ordinal score is never compared against a dichotomised one. Optimism-corrected values come from 1000 bootstrap resamples. The integrated discrimination improvement follows the Pencina definition with 1000-resample bootstrap percentile intervals; net reclassification indices are given in Supplementary Table S10. The extended reference model contains the arrival classification together with age, sex, comorbidity burden, lactate, oxygen saturation, heart rate, and temperature at presentation, and fits on the 1076 patients with a complete comorbidity count. AUC, area under the receiver operating characteristic curve; CI, confidence interval; df, degrees of freedom; qSOFA, quick Sequential Organ Failure Assessment.

## Data Availability

The data for this study were obtained from the same patient cohort as the residency (medical specialty) thesis of Ayşenur Özçelik, completed at the Department of Emergency Medicine, Bolu Abant İzzet Baysal University Faculty of Medicine. Patient identifiers were removed before any analysis, and the dataset was held within the institution throughout. No text, table, or figure is duplicated from either source. The data presented in this study are available on request from the corresponding author. The data are not publicly available because they consist of retrospectively collected clinical records for which individual informed consent was waived by the ethics committee; the approval (protocol code 2025/165, 22 April 2025) permits analysis of the de-identified dataset within the institution only, and any release to third parties requires separate institutional approval. The analysis code that produced every reported quantity is available from the corresponding author on request.

## Supplementary Materials

The following supporting information can be downloaded at https://www.mdpi.com/article/10.3390/jcm15197591/s1. Table S1. Missingness audit for all 48 variables entering any analysis (variable-level missingness within the 1077 analysed patients; patient-level exclusion during cohort assembly is described in Section 2.2 and Figure 1 of the main text); Table S2. Field-to-triage trajectory view of the same cohort (secondary framing); Table S3. Component-level analysis of the three prehospital qSOFA criteria; Table S4. Diagnostic accuracy of qSOFA of 2 or more for Sepsis-3. Predictive values at other prevalences are recalculated from the observed sensitivity and specificity and carry no confidence interval; Table S5. Pre-specified sensitivity analyses. Odds ratios are from logistic regression with pro-file-likelihood confidence intervals; multiply imputed estimates pool five imputations; Table S6. Comparison against previously published prehospital qSOFA studies. Reference numbers refer to the main-text reference list; Table S7. Multiplicity-adjusted *p* values for the twelve within-stratum contrasts (prehospital qSOFA ≥ 2 versus <2). Fisher exact *p* values are those of Table 2 in the main text; Holm and Bonferroni adjustments are applied across the five secondary outcomes within each stratum and across all twelve contrasts; Table S8. Within-stratum adjusted odds ratios for prehospital qSOFA ≥ 2 with the extended covariate set (age, sex, comorbidity burden, lactate, oxygen saturation, heart rate and temperature at ED presentation). The triage-negative models fit on 684 patients because one comorbidity count is missing; Table S9. Extended clinical reference model for 30-day mortality with and without the prehospital qSOFA classification. The reference model contains qSOFA ≥ 2 at ED triage, age, sex, comorbidity bur-den, lactate, oxygen saturation, heart rate and temperature; n = 1076. Confidence intervals for odds ratios are profile-likelihood intervals; AUC intervals are DeLong intervals; the integrated discrimination improvement carries a 1000-resample bootstrap percentile interval; net benefit follows Vickers and Elkin; Table S10. Net reclassification indices for adding the prehospital qSOFA classification to the ED-arrival classification. Indices follow the Pencina definitions with 1000-resample bootstrap percentile intervals. Because no accepted action threshold exists for this decision, the categorical index at any fixed pair of cut-offs is not the interpretable quantity; see main text Section 3.5; Table S11. Nested analysis sets, the variables each one requires, and its denominator; and the three reasons why eligible patients did not enter any analysis set. Percentages in the lower panel are of the 1481 excluded patients and of the 2558 eligible patients. ED, emergency department; GCS, Glasgow Coma Scale; qSOFA, quick Sequential Organ Failure Assessment; SOFA, Sequential Organ Failure Assessment. No patient contributing to the primary analysis was removed because a laboratory value was missing. No data on the 1481 excluded patients were retained in the study database; Table S12. The primary within-stratum contrast repeated within strata of emergency department disposition. Selection on the availability of an in-hospital record would be expected to act through disposition, so the association is examined separately in patients discharged from the department. CI, confi-dence interval; ED, emergency department; qSOFA, quick Sequential Organ Failure Assessment. Risk ratios are Katz log-transformed with exact (Fisher) *p* values. Of the 1077 analysed patients, 278 (25.8%) were discharged from the emergency department and their 30-day mortality was 10.1%. In the triage-negative stratum, the adjusted odds ratio among discharged patients was 2.90 (1.17 to 6.86) (*p* = 0.017; age, sex and comorbidity burden; n = 271). The interaction between the prehospital classification and disposition was 1.31 (0.52 to 3.22) (likelihood-ratio p = 0.559). The triage-positive discharged cell contains six patients and is shown for completeness only; Table S13. Quantitative bias analysis for cohort selection. Panel A converts the observed risk ratios into the minimum strength of selection that could explain them; Panel B returns the 1481 excluded patients to the cohort across 27 scenarios per stratum; Panel C gives a non-parametric envelope for the 266 transferred patients considered alone. CI, confidence interval; ED, emergency department; qSOFA, quick Sequential Organ Failure Assessment. Panel A follows Result 1B and Result 3B of Smith and VanderWeele [30]; each value is the minimum that every one of the parameters linking the un-measured selection factors to exposure and to outcome would have to attain. Panel B varies the share of the excluded patients falling in each stratum (0.40, 0.636, 0.85), the prevalence of a positive prehospital classification among them (0.5, 1.0 and 1.5 times the observed prevalence) and their mortality level (0.25, 0.5 and 1.0 times the observed level); the base case is the middle value of each. In the four scenarios in which the triage-positive estimate is nullified by a positive association, the required risk ratio is 1.03 to 1.49, and all four combine a prehospital-positive prevalence 1.5 times the observed with mortality at a quarter to a half of the observed level. Panel C assigns every unobserved outcome among the transferred patients in the direction that minimises and then maximises the risk ratio, so the envelope is not a confidence interval and is expected to include 1.

## Abbreviations

The following abbreviations are used in this manuscript:

AIC	Akaike information criterion
AUC	area under the receiver operating characteristic curve
CI	confidence interval
ED	emergency department
EMS	emergency medical services
GCS	Glasgow Coma Scale
ICU	intensive care unit
IDI	integrated discrimination improvement
IQR	interquartile range
MEWS	Modified Early Warning Score
NEWS	National Early Warning Score
NRI	net reclassification improvement
qSOFA	quick Sequential Organ Failure Assessment
REMS	Rapid Emergency Medicine Score
SIRS	systemic inflammatory response syndrome
SOFA	Sequential Organ Failure Assessment
STARD	Standards for Reporting Diagnostic Accuracy Studies
STROBE	Strengthening the Reporting of Observational Studies in Epidemiology
