# Supplementary material for: Prehospital qSOFA Is Associated with 30-Day Mortality Within Emergency Department Triage Strata in Ambulance-Transported Adults with Suspected Infection: An Exploratory Three-Year Retrospective Cohort Study
Source: J Clin Med. 2026 Sep 30;15(19):7591. doi: 10.3390/jcm15197591 (PMC13666278; doi:10.3390/jcm15197591)
Supplement: Supplementary file 1 [file jcm-15-07591-s001.zip › jcm-4572261-supplementary.pdf]

Journal of
Clinical Medicine

MDPI

Supplementary Materials

Prehospital qSOFA Is Associated with 30-Day Mortality Within Emergency Department Triage Strata in Ambulance-Transported Adults with Suspected Infection: An Exploratory Three-Year Retrospective Cohort Study

Table S1. Missingness audit for all 48 variables entering any analysis (variable-level missingness within the 1,077 analysed patients; patient-level exclusion during cohort assembly is described in Section 2.2 and Figure 1 of the main text).

Variable	Non-missing	Missing	Missing, %
age	1077	0	0.00
male	1077	0	0.00
comorb_burden	1076	1	0.09
cm_HT	1076	1	0.09
cm_DM	1077	0	0.00
cm_KAH	1077	0	0.00
cm_KOAH	1077	0	0.00
cm_MALIG	1077	0	0.00
cm_KY	1077	0	0.00
cm_KBY	1077	0	0.00
cm_KCS	1077	0	0.00
cm_SVO	1077	0	0.00
rr_ed	1077	0	0.00
sbp_ed	1077	0	0.00
dbp_ed	1077	0	0.00
map_ed	1077	0	0.00
gcs_raw	1077	0	0.00
gcs_cont	1075	2	0.19
pulse	1077	0	0.00
temp	1077	0	0.00
spo2	1077	0	0.00
lactate	1077	0	0.00
sofa	1077	0	0.00
creat	1077	0	0.00
plt	1077	0	0.00
bili	1077	0	0.00
pf	1077	0	0.00
wbc	1077	0	0.00
crp	1074	3	0.28
ph_resp	1077	0	0.00
ph_ment	1077	0	0.00
ph_sbp	1077	0	0.00
ph_sbp_crit	1077	0	0.00
ph_qs	1077	0	0.00
ed_qs	1077	0	0.00
ph_hi	1077	0	0.00
ed_hi	1077	0	0.00
stratum	1077	0	0.00
cell	1077	0	0.00
mort30	1077	0	0.00
intub	1077	0	0.00

vaso_ed	1077	0	0.00
vaso_wd	1077	0	0.00
icu	1077	0	0.00
organ_support	1077	0	0.00
los	1077	0	0.00
sepsis3	1077	0	0.00
dispo	1077	0	0.00

Table S2. Field-to-triage trajectory view of the same cohort (secondary framing).

Trajectory group	n	30-day mortality, % (95% CI)	Composite organ support, % (95% CI)	Adjusted odds ratio for death (95% CI) vs stable low
Stable low: prehospital <2, triage <2	554	13.9 (11.3–17.0)	21.7 (18.4–25.3)	reference
Converted: prehospital <2, triage ≥2	198	40.4 (33.8–47.4)	81.3 (75.3–86.1)	4.105 (2.822–5.989)
Reverted: prehospital ≥2, triage <2	131	26.0 (19.2–34.1)	40.5 (32.4–49.0)	2.115 (1.323–3.335)
Stable high: prehospital ≥2, triage ≥2	194	55.2 (48.1–62.0)	91.2 (86.4–94.5)	7.376 (5.083–10.782)

Table S3. Component-level analysis of the three prehospital qSOFA criteria.

Prehospital component	Prevalence, %	Mortality if met, %	Mortality if not met, %	AUC (95% CI)	Adjusted odds ratio (95% CI)	p Value
Respiratory criterion met	44.6	32.1	24.1	0.549 (0.516–0.582)	1.475 (1.126–1.935)	0.005
Altered mentation criterion met	24.9	35.4	25.1	0.548 (0.518–0.579)	1.535 (1.135–2.070)	0.005
Systolic blood pressure ≤100 mmHg	39.5	39.3	20.1	0.615 (0.582–0.647)	2.547 (1.937–3.357)	<0.001

Table S4. Diagnostic accuracy of qSOFA of 2 or more for Sepsis-3. Predictive values at other prevalences are recalculated from the observed sensitivity and specificity and carry no confidence interval.

Measure	Prehospital qSOFA ≥2 (95% CI)	qSOFA ≥2 at triage (95% CI)
Sensitivity, %	34.4 (31.3–37.5)	43.4 (40.2–46.7)
Specificity, %	90.6 (85.5–94.1)	98.3 (95.2–99.4)
Positive predictive value, %	94.8 (91.8–96.7)	99.2 (97.8–99.7)
Negative predictive value, %	21.8 (19.0–24.9)	26.0 (22.8–29.4)
Positive likelihood ratio	3.66 (2.31–5.81)	26.19 (8.51–80.66)
Negative likelihood ratio	0.72 (0.68–0.77)	0.58 (0.54–0.61)
Accuracy, %	43.8	52.6
Positive predictive value at 5% prevalence, %	16.2	58.0
Positive predictive value at 10% prevalence, %	28.9	74.4
Positive predictive value at 20% prevalence, %	47.8	86.8

Negative predictive value at 10% prevalence, %	92.6	94.0

Table S5. Pre-specified sensitivity analyses. Odds ratios are from logistic regression with profile-likelihood confidence intervals; multiply imputed estimates pool five imputations.

Sensitivity analysis	Model, term or subgroup	Estimate (95% CI)	p Value
Adjusted OR for 30-day mortality, interaction model, complete case	qSOFA ≥2 at ED triage	4.105 (2.822–5.989)	<0.001
Adjusted OR for 30-day mortality, interaction model, complete case	Prehospital qSOFA ≥2	2.115 (1.323–3.335)	0.001
Adjusted OR for 30-day mortality, interaction model, complete case	Age, per year	1.011 (0.999–1.023)	0.073
Adjusted OR for 30-day mortality, interaction model, complete case	Male sex	0.990 (0.739–1.326)	0.945
Adjusted OR for 30-day mortality, interaction model, complete case	Comorbidity burden, per condition	0.936 (0.843–1.038)	0.213
Adjusted OR for 30-day mortality, interaction model, complete case	Triage × prehospital interaction	0.850 (0.462–1.576)	0.602
Adjusted OR for 30-day mortality, interaction model, multiply imputed (m = 5)	qSOFA ≥2 at ED triage	4.109 (2.821–5.986)	<0.001
Adjusted OR for 30-day mortality, interaction model, multiply imputed (m = 5)	Prehospital qSOFA ≥2	2.117 (1.334–3.360)	0.001
Adjusted OR for 30-day mortality, interaction model, multiply imputed (m = 5)	Age, per year	1.011 (0.999–1.023)	0.069
Adjusted OR for 30-day mortality, interaction model, multiply imputed (m = 5)	Male sex	0.989 (0.739–1.325)	0.943
Adjusted OR for 30-day mortality, interaction model, multiply imputed (m = 5)	Comorbidity burden, per condition	0.936 (0.844–1.039)	0.217
Adjusted OR for 30-day mortality, interaction model, multiply imputed (m = 5)	Triage × prehospital interaction	0.849 (0.460–1.567)	0.599
Prehospital qSOFA >= 2 adjusted OR for 30-day mortality within the stratum, multiply imputed	A: ED qSOFA < 2	2.057 (1.291–3.278)	0.002
Prehospital qSOFA >= 2 adjusted OR for 30-day mortality within the stratum, multiply imputed	B: ED qSOFA >= 2	1.839 (1.227–2.757)	0.003
Adjusted OR for 30-day mortality per one-point increase in the prehospital qSOFA score, within the stratum	A: ED qSOFA < 2	1.456 (1.118–1.898)	0.005
30-day mortality at prehospital qSOFA = 0 within the stratum, %	A: ED qSOFA < 2 | prehospital score 0	12.7 (9.0–17.5)	
30-day mortality at prehospital qSOFA = 1 within the stratum, %	A: ED qSOFA < 2 | prehospital score 1	14.8 (11.3–19.2)	
30-day mortality at prehospital qSOFA = 2 within the stratum, %	A: ED qSOFA < 2 | prehospital score 2	26.1 (19.0–34.6)	
30-day mortality at prehospital qSOFA = 3 within the stratum, %	A: ED qSOFA < 2 | prehospital score 3	25.0 (8.9–53.2)	
Trend in 30-day mortality across the prehospital qSOFA score within the stratum	A: ED qSOFA < 2		0.002
Adjusted OR for 30-day mortality per one-point increase in the prehospital qSOFA score, within the stratum	B: ED qSOFA >= 2	1.300 (1.043–1.628)	0.021
30-day mortality at prehospital qSOFA = 0 within the stratum, %	B: ED qSOFA >= 2 | prehospital score 0	45.8 (33.7–58.3)	
30-day mortality at prehospital qSOFA = 1 within the stratum, %	B: ED qSOFA >= 2 | prehospital score 1	38.1 (30.5–46.4)	

30-day mortality at prehospital qSOFA = 2 within the stratum, %	B: ED qSOFA >= 2 ∣ prehospital score 2	54.7 (46.4–62.7)	
30-day mortality at prehospital qSOFA = 3 within the stratum, %	B: ED qSOFA >= 2 ∣ prehospital score 3	56.4 (43.3–68.6)	
Trend in 30-day mortality across the prehospital qSOFA score within the stratum	B: ED qSOFA >= 2		0.024
Adjusted OR for 30-day mortality per qSOFA point, both scores ordinal	ED-arrival qSOFA, per point	2.330 (1.965–2.776)	<0.001
Adjusted OR for 30-day mortality per qSOFA point, both scores ordinal	Prehospital qSOFA, per point	1.224 (1.026–1.460)	0.025
Adjusted OR for 30-day mortality per qSOFA point, both scores ordinal	Age, per year	1.009 (0.997–1.021)	0.154
Adjusted OR for 30-day mortality per qSOFA point, both scores ordinal	Male sex	0.941 (0.701–1.263)	0.683
Adjusted OR for 30-day mortality per qSOFA point, both scores ordinal	Comorbidity burden, per condition	0.940 (0.846–1.043)	0.246
30-day mortality at Prehospital qSOFA = 0, %	Prehospital qSOFA = 0	19.3 (15.2–24.1)	
30-day mortality at Prehospital qSOFA = 1, %	Prehospital qSOFA = 1	21.9 (18.4–26.0)	
30-day mortality at Prehospital qSOFA = 2, %	Prehospital qSOFA = 2	41.5 (35.6–47.6)	
30-day mortality at Prehospital qSOFA = 3, %	Prehospital qSOFA = 3	50.7 (39.1–62.3)	
Trend in 30-day mortality across Prehospital qSOFA 0-3	Prehospital qSOFA		<0.001
30-day mortality at ED-arrival qSOFA = 0, %	ED-arrival qSOFA = 0	9.9 (7.1–13.8)	
30-day mortality at ED-arrival qSOFA = 1, %	ED-arrival qSOFA = 1	21.4 (17.6–25.9)	
30-day mortality at ED-arrival qSOFA = 2, %	ED-arrival qSOFA = 2	41.8 (36.2–47.6)	
30-day mortality at ED-arrival qSOFA = 3, %	ED-arrival qSOFA = 3	63.6 (54.1–72.1)	
Trend in 30-day mortality across ED-arrival qSOFA 0-3	ED-arrival qSOFA		<0.001
Sepsis-3-positive subgroup size	Sepsis-3 positive	896	
30-day mortality, Sepsis-3-positive only, %	A: ED qSOFA < 2 ∣ prehospital qSOFA < 2	17.3 (13.9–21.4)	
30-day mortality, Sepsis-3-positive only, %	A: ED qSOFA < 2 ∣ prehospital qSOFA >= 2	27.0 (19.7–35.7)	
30-day mortality risk difference, Sepsis-3-positive only, percentage points	A: ED qSOFA < 2	9.6 (0.9–19.5)	
Prehospital qSOFA >= 2 adjusted OR for 30-day mortality, Sepsis-3-positive only	A: ED qSOFA < 2	1.717 (1.040–2.794)	0.032
30-day mortality, Sepsis-3-positive only, %	B: ED qSOFA >= 2 ∣ prehospital qSOFA < 2	40.8 (34.2–47.8)	
30-day mortality, Sepsis-3-positive only, %	B: ED qSOFA >= 2 ∣ prehospital qSOFA >= 2	54.9 (47.9–61.8)	
30-day mortality risk difference, Sepsis-3-positive only, percentage points	B: ED qSOFA >= 2	14.1 (3.8–24.0)	
Prehospital qSOFA >= 2 adjusted OR for 30-day mortality, Sepsis-3-positive only	B: ED qSOFA >= 2	1.793 (1.199–2.694)	0.005
ED x prehospital interaction odds ratio, Sepsis-3-positive only	Triage × prehospital interaction	1.013 (0.539–1.922)	0.968
Likelihood-ratio test for the interaction term, Sepsis-3-positive only	interaction vs main effects	$\chi^2 = 0.002$	0.968
Likelihood-ratio test for adding the prehospital value, Sepsis-3-positive only	M1 -> M2, dichotomised	$\chi^2 = 12.718$	<0.001
AUC for 30-day mortality, Sepsis-3-positive only	M1: ED-arrival qSOFA >= 2 alone, Sepsis-3-positive	0.66 (0.63–0.69)	
AUC for 30-day mortality, Sepsis-3-positive only	M2: ED-arrival + prehospital qSOFA >= 2, Sepsis-3-positive	0.69 (0.65–0.72)	

Pre-specified sensitivity analysis excluding patients with treatment-limitation orders	not performed (variable not recorded)
Time-to-event (survival) analysis of 30-day mortality	not performed (variable not recorded)

Table S6. Comparison against previously published prehospital qSOFA studies. Reference numbers refer to the main-text reference list.

First author and year	Country and setting	Design	n	Population	Outcome assessed	Reported sensitivity and specificity, or AUC	qSOFA measured once or at two time points	Source of the reported values	Reference number
Dorsett et al., 2017	USA; one urban EMS system and its receiving emergency department	Retrospective cohort	152	Emergency department patients with confirmed or suspected infection transported by EMS (infection without sepsis 71, sepsis 38, severe sepsis or septic shock 43)	Severe sepsis or septic shock confirmed in the emergency department	Field qSOFA 2 or higher: sensitivity 16.3% (95% CI 6.8-30.7), specificity 97.3% (95% CI 92.1-99.4). During the emergency department stay sensitivity 67.4% (95% CI 51.5-80.9)	Two (field and emergency department)	Structured abstract (full text not obtainable)	[11]
Tusgul et al., 2017	Switzerland; Lausanne prehospital system and university emergency department	Retrospective cohort	886	Ambulance-transported patients with suspected infection	Intensive care admission; 48-hour mortality	Prehospital qSOFA 2 or higher: sensitivity 36.3% for intensive care admission and 68.0% for 48-hour mortality; at emergency department triage 31.2% and 60.0%	Two (field and emergency department triage)	Full text	[12]
Koyama et al., 2019	Japan; one tertiary emergency department	Retrospective cohort	925	Emergency department patients with suspected infection transported by ambulance	In-hospital mortality	In-hospital mortality 14.0% (66/473) in field qSOFA-positive vs 6.0% (27/452) in field qSOFA-	One (field)	Full text	[18]

						negative patients; adjusted HR 2.41 (95% CI 1.51-3.98). Discrimination statistics not reported			
Lane et al., 2020	Canada; province-wide paramedic records linked to hospital databases, Ontario	Retrospective validation study	131,745	Adults transported by paramedics and linked to hospital records	Sepsis diagnosed in the emergency department	qSOFA at threshold 2: sensitivity 0.40 (95% CI 0.38-0.42), specificity 0.94 (95% CI 0.94-0.95); across all 21 strategies sensitivity ranged 0.02-0.85 and specificity 0.38-0.99	One (field)	Full text (Table 3)	[13]
Miyamoto et al., 2020	Japan; nationwide trauma registry	Retrospective registry analysis	90,974	Trauma patients transported to hospital	In-hospital mortality	Field qSOFA 0 rising to 2 or higher on hospital arrival: adjusted OR 3.46 (95% CI 2.41-4.85). Sensitivity and specificity for qSOFA alone not reported	Two (field and hospital arrival)	Full text	[23]
Lindskou et al., 2023	Denmark; regional ambulance service, North Denmark Region	Retrospective prognostic cohort	107,569	Unique patients transported by ambulance, all causes	30-day mortality	qSOFA AUROC 0.59 (95% CI 0.59-0.60), below NEWS2, mNEWS, RETTS and DEPT (AUROC 0.67-0.68)	One (field)	Full text	[15]
Saito et al., 2023	Japan; one tertiary emergency department	Retrospective cohort	2,407	Ambulance-transported adults with suspected infection	Sepsis by the Sepsis-3 definition	Prehospital qSOFA c-statistic 0.65 (95% CI 0.62-0.68) vs	Two (field and emergency department)	Full text	[26]

						emergency department qSOFA 0.67 (95% CI 0.64-0.70), p = 0.15; sensitivity 0.57 (95% CI 0.52-0.62) vs 0.55 (95% CI 0.50-0.60). The combined field-and-department score identified 44 of 369 sepsis cases (12%) missed by the department score alone			
Piedmont et al., 2024	Germany; emergency medical services of one federal state	Retrospective cohort	221,429	Prehospital medical records of adult emergency patients	Sepsis diagnosed in hospital	qSOFA sensitivity 23.1% (95% CI 21.8-24.3), specificity 96.6%; NEWS2 sensitivity 73.1% (95% CI 71.8-74.4), AUROC 0.77 (95% CI 0.71-0.83)	One (field)	Full text	[10]
Goodacre et al., 2024	England; two ambulance services and four acute hospitals	Retrospective diagnostic cohort with decision-analytic modelling	95,022 episodes (71,204 patients); 12,870 episodes in the diagnostic accuracy cohort	Adults transported to hospital by emergency ambulance, excluding injury, mental health, cardiac arrest and transfers	Sepsis by the Sepsis-3 definition receiving urgent treatment	qSOFA above 1 applied to a paramedic impression of sepsis or infection: sensitivity 0.305 (95% CI 0.259-0.355), positive predictive value 0.356 (95% CI 0.304-0.412). Best overall strategy	One (field)	Full text	[14]

						was NEWS2 on the same impression, AUROC 0.756 (95% CI 0.729-0.783); no strategy reached sensitivity above 0.8 with positive predictive value above 0.15			
Andersson et al., 2026	Norway; ambulance service with in-hospital follow-up	Prospective cohort	398	Prehospital patients with suspected sepsis (median age 76 years)	Sepsis severity by SOFA score; septic shock; 30-day all-cause mortality (10.1%)	Area under the curve for septic shock higher for qSOFA than for NEWS2 (p = 0.033); absolute values not given in the abstract	One (field)	Structured abstract (full text not obtainable)	[33]
Present study, 2026	Turkiye; one university hospital emergency department receiving 112 EMS transports	Retrospective single-centre cohort	1,077	Adults transported by 112 emergency medical services with already-suspected infection, 2022-2024 (median age 77 years)	30-day mortality; intubation, vasopressor use, ICU admission, composite organ support	Among patients qSOFA-negative at ED triage, 30-day mortality 26.0% if field-positive vs 13.9% if field-negative (adjusted OR 2.06, 95% CI 1.28–3.25); among triage-positive patients 55.2% vs 40.4% (adjusted OR 1.84, 1.23–2.76). AUC 0.682 to 0.713 when the field value is added to	Two (field and emergency department triage)	Present study	-

the arrival value alone; 0.751 to 0.758 when added to an extended clinical model

Table S7. Multiplicity-adjusted p values for the twelve within-stratum contrasts (prehospital qSOFA ≥2 versus <2). Fisher exact p values are those of Table 2 in the main text; Holm and Bonferroni adjustments are applied across the five secondary outcomes within each stratum and across all twelve contrasts.

Triage stratum	Outcome	Fisher exact p (unadjusted)	Holm-adjusted p, five secondary outcomes within stratum	Holm-adjusted p, all twelve contrasts	Bonferroni-adjusted p, all twelve contrasts
qSOFA <2 at ED triage (n = 685)	30-day mortality	0.001	primary outcome	0.010	0.017
qSOFA <2 at ED triage (n = 685)	Intubation	<0.001	0.001	0.003	0.004
qSOFA <2 at ED triage (n = 685)	Vasopressor use in the ED	0.013	0.013	0.026	0.159
qSOFA <2 at ED triage (n = 685)	Vasopressor use on the ward	0.005	0.009	0.023	0.054
qSOFA <2 at ED triage (n = 685)	ICU admission	<0.001	<0.001	<0.001	<0.001
qSOFA <2 at ED triage (n = 685)	Composite organ support	<0.001	<0.001	<0.001	<0.001
qSOFA ≥2 at ED triage (n = 392)	30-day mortality	0.005	primary outcome	0.023	0.055
qSOFA ≥2 at ED triage (n = 392)	Intubation	<0.001	<0.001	<0.001	<0.001
qSOFA ≥2 at ED triage (n = 392)	Vasopressor use in the ED	0.002	0.006	0.012	0.025
qSOFA ≥2 at ED triage (n = 392)	Vasopressor use on the ward	<0.001	<0.001	<0.001	<0.001
qSOFA ≥2 at ED triage (n = 392)	ICU admission	0.013	0.013	0.026	0.159
qSOFA ≥2 at ED triage (n = 392)	Composite organ support	0.005	0.010	0.023	0.062

Table S8. Within-stratum adjusted odds ratios for prehospital qSOFA ≥2 with the extended covariate set (age, sex, comorbidity burden, lactate, oxygen saturation, heart rate and temperature at ED presentation). The triage-negative models fit on 684 patients because one comorbidity count is missing.

Triage stratum	Outcome	Events, n	Adjusted odds ratio for prehospital qSOFA ≥2 (95% CI)	p Value
qSOFA <2 at ED triage (n = 684)	30-day mortality	111	1.76 (1.07–2.83)	0.023

qSOFA <2 at ED triage (n = 684)	Intubation	12	7.02 (1.78–31.09)	0.006
qSOFA <2 at ED triage (n = 684)	Vasopressor use in the ED	15	3.58 (1.08–11.30)	0.030
qSOFA <2 at ED triage (n = 684)	Vasopressor use on the ward	95	1.61 (0.94–2.71)	0.076
qSOFA <2 at ED triage (n = 684)	ICU admission	164	1.91 (1.22–2.97)	0.004
qSOFA <2 at ED triage (n = 684)	Composite organ support	173	2.00 (1.29–3.09)	0.002
qSOFA ≥2 at ED triage (n = 392)	30-day mortality	187	1.65 (1.08–2.54)	0.021
qSOFA ≥2 at ED triage (n = 392)	Intubation	117	3.16 (1.90–5.37)	<0.001
qSOFA ≥2 at ED triage (n = 392)	Vasopressor use in the ED	104	1.83 (1.12–3.02)	0.017
qSOFA ≥2 at ED triage (n = 392)	Vasopressor use on the ward	251	2.22 (1.42–3.47)	<0.001
qSOFA ≥2 at ED triage (n = 392)	ICU admission	320	1.65 (0.96–2.89)	0.075
qSOFA ≥2 at ED triage (n = 392)	Composite organ support	338	1.79 (0.93–3.51)	0.085

Table S9. Extended clinical reference model for 30-day mortality with and without the prehospital qSOFA classification. The reference model contains qSOFA ≥2 at ED triage, age, sex, comorbidity burden, lactate, oxygen saturation, heart rate and temperature; n = 1,076. Confidence intervals for odds ratios are profile-likelihood intervals; AUC intervals are DeLong intervals; the integrated discrimination improvement carries a 1,000-resample bootstrap percentile interval; net benefit follows Vickers and Elkin.

Measure	Reference model without prehospital qSOFA	Reference model with prehospital qSOFA	Difference or test result
Complete-case n			1076
Odds ratio, qSOFA ≥2 at ED triage (95% CI), model with prehospital qSOFA		3.079 (2.237–4.246)	<0.001
Odds ratio, Age, per year (95% CI), model with prehospital qSOFA		1.012 (1.000–1.025)	0.050
Odds ratio, Male sex (95% CI), model with prehospital qSOFA		0.989 (0.733–1.336)	0.943
Odds ratio, Comorbidity burden, per condition (95% CI), model with prehospital qSOFA		0.929 (0.834–1.033)	0.178
Odds ratio, Lactate, per mmol/L (95% CI), model with prehospital qSOFA		1.289 (1.178–1.419)	<0.001
Odds ratio, Oxygen saturation, per % (95% CI), model with prehospital qSOFA		0.984 (0.969–0.999)	0.040
Odds ratio, Heart rate, per beat/min (95% CI), model with prehospital qSOFA		0.999 (0.995–1.002)	0.491
Odds ratio, Temperature, per °C (95% CI), model with prehospital qSOFA		0.965 (0.884–1.010)	0.289
Odds ratio, prehospital qSOFA ≥2 (95% CI)	not in model	1.709 (1.243–2.342)	<0.001
Akaike information criterion	1112.7	1103.9	-8.8
Likelihood-ratio test for adding the prehospital value			χ^2 = 10.81, 1 df, p = 0.001

AUC (95% CI)	0.751 (0.719–0.783)	0.758 (0.726–0.789)	+0.007, DeLong p = 0.23
Optimism-corrected AUC (1,000 bootstrap resamples)	0.743	0.749	
Brier score	0.168	0.166	
Integrated discrimination improvement (95% CI)			0.010 (0.001–0.024)
Net benefit at a threshold probability of 15%	0.165	0.171	+0.007
Net benefit at a threshold probability of 25%	0.114	0.114	+0.001
Net benefit at a threshold probability of 40%	0.047	0.061	+0.015
Clinical covariates only, no qSOFA term: AUC (95% CI)			0.707 (0.672–0.741)
Ordinal variant (both scores 0 to 3)			
Odds ratio, prehospital qSOFA per point (95% CI)	not in model	1.182 (0.986–1.418)	0.071
Likelihood-ratio test for adding the prehospital score			χ^2 = 3.26, 1 df, p = 0.071
AUC (95% CI)	0.757 (0.725–0.789)	0.760 (0.728–0.791)	+0.003, DeLong p = 0.32
Triage × prehospital interaction, extended covariates (95% CI)			0.917 (0.489–1.737); LRT χ^2 = 0.072, p = 0.79
Implied prehospital odds ratio within the triage-negative stratum, interaction model (95% CI)			1.80 (1.11–2.90)
Implied prehospital odds ratio within the triage-positive stratum, interaction model (95% CI)			1.65 (1.09–2.50)

Table S10. Net reclassification indices for adding the prehospital qSOFA classification to the ED-arrival classification. Indices follow the Pencina definitions with 1,000-resample bootstrap percentile intervals. Because no accepted action threshold exists for this decision, the categorical index at any fixed pair of cut-offs is not the interpretable quantity; see main text Section 3.5.

Index	Value (95% CI)
Continuous NRI, two-score comparison (dichotomised)	0.474 (0.342–0.599)
Categorical NRI, cut-offs 10% and 30%	0.000 (0.000–0.000)
Categorical NRI, cut-offs 20% and 40%	-0.127 (-0.153–0.012)
Integrated discrimination improvement, two-score comparison	0.017 (0.004–0.036)
Predicted 30-day risks, arrival-only model (dichotomised)	0.162 (triage-negative) and 0.477 (triage-positive)
Predicted 30-day risks, two-variable model (dichotomised)	0.142, 0.245, 0.395 and 0.561 for the four cells
Reason the categorical NRI at 10% and 30% is exactly zero	Each of the four two-variable risks falls in the same 10-to-30% or above-30% band as its arrival-only counterpart, so no patient crosses a boundary and all four reclassification counts are zero

Table S11. Nested analysis sets, the variables each one requires, and its denominator; and the three reasons why eligible patients did not enter any analysis set. Percentages in the lower panel are of the 1,481 excluded patients and of the 2,558 eligible patients. ED, emergency department; GCS, Glasgow Coma Scale; qSOFA, quick Sequential Organ Failure Assessment; SOFA, Sequential Organ Failure Assessment. No patient contributing to the primary analysis was removed because a laboratory value was missing. No data on the 1,481 excluded patients were retained in the study database.

**Analysis set**	**Variables required in addition to the preceding set**	**n**	**Not available**

Primary within-stratum contrast (risk differences, risk ratios, Fisher tests)	Prehospital qSOFA classification, ED-arrival qSOFA classification, 30-day vital status	1,077	0
Primary adjusted models and the interaction test	Age, sex, comorbidity burden	1,076	1 (comorbidity burden not recorded)
SOFA-based analyses and the Sepsis-3 reference standard	SOFA score and its components	1,077	0
Extended clinical reference model	Lactate, oxygen saturation, heart rate, temperature	1,076	1 (comorbidity burden not recorded)
Continuous-GCS sensitivity analysis	Glasgow Coma Scale as a continuous value	1,075	2 (value outside 3 to 15)
Descriptive C-reactive protein	C-reactive protein	1,074	3 (not measured)
**Reason an eligible patient entered no analysis set**		n (% of excluded; % of eligible)	
Hospital episode record could not be retrieved from the information system or had not been completed; arrival vital signs, disposition and 30-day vital status all absent		1,175 (79.3; 45.9)	
Transferred to another institution before an outcome could be ascertained		266 (18.0; 10.4)	
Left the emergency department before evaluation; no arrival measurement		40 (2.7; 1.6)	
**Total excluded**		1,481 (100.0; 57.9)	

Table S12. The primary within-stratum contrast repeated within strata of emergency department disposition. Selection on the availability of an in-hospital record would be expected to act through disposition, so the association is examined separately in patients discharged from the department. CI, confidence interval; ED, emergency department; qSOFA, quick Sequential Organ Failure Assessment. Risk ratios are Katz log-transformed with exact (Fisher) p values. Of the 1,077 analysed patients, 278 (25.8%) were discharged from the emergency department and their 30-day mortality was 10.1%. In the triage-negative stratum, the adjusted odds ratio among discharged patients was 2.90 (1.17 to 6.86) ($p = 0.017$; age, sex and comorbidity burden; $n = 271$). The interaction between the prehospital classification and disposition was 1.31 (0.52 to 3.22) (likelihood-ratio $p = 0.559$). The triage-positive discharged cell contains six patients and is shown for completeness only.

ED disposition	ED triage qSOFA stratum	Prehospital qSOFA <2, deaths/n (%)	Prehospital qSOFA ≥2, deaths/n (%)	Risk ratio (95% CI)	p
Discharged from the ED	<2	17/225 (7.6)	10/47 (21.3)	2.82 (1.38 to 5.76)	0.012
Discharged from the ED	≥2	1/2 (50.0)	0/4 (0.0)	Not estimated (n = 6)	-
Admitted or died in the ED	<2	60/329 (18.2)	24/84 (28.6)	1.57 (1.04 to 2.36)	0.047
Admitted or died in the ED	≥2	79/196 (40.3)	107/190 (56.3)	1.40 (1.13 to 1.73)	0.002

Table S13. Quantitative bias analysis for cohort selection. Panel A converts the observed risk ratios into the minimum strength of selection that could explain them; Panel B returns the 1,481 excluded patients to the cohort across 27 scenarios per stratum; Panel C gives a non-parametric envelope for the 266 transferred patients considered alone. CI, confidence interval; ED, emergency department; qSOFA, quick Sequential Organ Failure Assessment. Panel A follows Result 1B and Result 3B of Smith and VanderWeele [30]; each value is the minimum that every one of the parameters linking the unmeasured selection factors to exposure and to outcome would have to attain. Panel B varies the share of the excluded patients falling in each stratum (0.40, 0.636, 0.85), the prevalence of a positive prehospital classification among them (0.5, 1.0 and 1.5 times the observed prevalence) and their mortality level (0.25, 0.5 and 1.0 times the observed level); the base case is the middle value of each. In the four scenarios in which the triage-positive estimate is nullified by a positive association, the required risk ratio is 1.03 to 1.49, and all four combine a prehospital-positive prevalence 1.5 times the observed with mortality at a quarter to a half of the observed level. Panel C assigns every unobserved outcome among the transferred patients in the direction that minimises and then maximises the risk ratio, so the envelope is not a confidence interval and is expected to include 1.

Quantity	ED triage qSOFA <2 (stratum A)	ED triage qSOFA ≥2 (stratum B)
**Panel A. Selection-bias summary measures (Smith and VanderWeele)**		
Observed risk ratio (95% CI)	1.867 (1.308 to 2.665)	1.365 (1.105 to 1.687)
Assumption-free bound, at the point estimate	2.07	1.61
Assumption-free bound, at the lower confidence limit	1.55	1.28
Bound when selection is associated with increased outcome risk in both exposure groups, at the point estimate	3.14	2.07
The same bound at the lower confidence limit	1.94	1.45
**Panel B. Tipping-point analysis, 1,481 excluded patients returned**		

Risk ratio required among the excluded patients to bring the pooled estimate to 1, base case	0.37	0.73
Scenarios in which no value can bring the pooled estimate to 1	17 of 27	10 of 27
Range of the required risk ratio across the remaining scenarios	0.06 to 0.69	0.36 to 1.49
Scenarios nullified by a positive association among the excluded patients	0 of 27	4 of 27
Pooled risk ratio if the excluded patients showed the same association as the analysed patients, across the grid	1.57 to 2.42	0.96 to 1.95
**Panel C. Transferred patients considered alone (n = 266)**		
Non-parametric envelope for the within-stratum risk ratio	0.67 to 3.64	0.85 to 1.98
